# Diesel exhaust particles alter gut microbiome and gene expression in the bumblebee *Bombus terrestris*

**DOI:** 10.1002/ece3.10180

**Published:** 2023-06-21

**Authors:** Dimitri Seidenath, Alfons R. Weig, Andreas Mittereder, Thomas Hillenbrand, Dieter Brüggemann, Thorsten Opel, Nico Langhof, Marcel Riedl, Heike Feldhaar, Oliver Otti

**Affiliations:** ^1^ Animal Ecology I, Bayreuth Center of Ecology and Environmental Research (BayCEER) University of Bayreuth Bayreuth Germany; ^2^ Keylab Genomics and Bioinformatics, Bayreuth Center of Ecology and Environmental Research (BayCEER) University of Bayreuth Bayreuth Germany; ^3^ Department of Engineering Thermodynamics and Transport Processes University of Bayreuth Bayreuth Germany; ^4^ Department of Ceramic Materials Engineering University of Bayreuth Bayreuth Germany; ^5^ Applied Zoology TU Dresden Dresden Germany

**Keywords:** air pollution, brake dust, insect decline, particulate matter, pollinator, transcriptome

## Abstract

Insect decline is a major threat to ecosystems around the world as they provide many important functions, such as pollination or pest control. Pollution is one of the main reasons for the decline, alongside changes in land use, global warming, and invasive species. While negative impacts of pesticides are well‐studied, there is still a lack of knowledge about the effects of other anthropogenic pollutants, such as airborne particulate matter, on insects. To address this, we exposed workers of the bumblebee *Bombus terrestris* to sublethal doses of diesel exhaust particles (DEPs) and brake dust, orally or via air. After 7 days, we looked at the composition of the gut microbiome and tracked changes in gene expression. While there were no changes in the other treatments, oral DEP exposure significantly altered the structure of the gut microbiome. In particular, the core bacterium *Snodgrassella* had a decreased abundance in the DEP treatment. Similarly, transcriptome analysis revealed changes in gene expression after oral DEP exposure, but not in the other treatments. The changes are related to metabolism and signal transduction, which indicates a general stress response. Taken together, our results suggest potential health effects of DEP exposure on insects, here shown in bumblebees, as gut dysbiosis may increase the susceptibility of bumblebees to pathogens, while a general stress response may lower available energy resources. Those effects may exacerbate under natural conditions where insects face a multiple‐stressor environment.

## Graphical abstract

Here we studied if airborne particulate matter from traffic is a potential driver of insect decline. We exposed workers of the bumblebee *Bombus terrestris* to sublethal doses of diesel exhaust particles and brake dust, orally or via air. Oral exposure to diesel exhaust particles changed microbiome composition and altered gene expression.

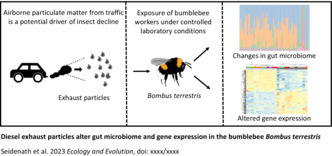

## 1 INTRODUCTION

Global biodiversity loss is one of the major challenges humanity currently faces (Díaz et al., 2006; Dirzo et al., 2014). Especially the rapid decline in insects is cause for concern, as they provide or contribute to many important ecosystem functions such as pollination, nutrient cycling, pest control, and linking trophic levels (Cardoso et al., 2020; Noriega et al., 2018). Pollution is one of the major reasons for the decline alongside intensification of land use, climate change, and invasive species (Miličić et al., 2021; Sánchez‐Bayo & Wyckhuys, 2019).

Pesticides harm insects on many different levels ranging from subtle changes in the gut microbiome over behavioral changes to increased mortality (Desneux et al., 2007; Motta et al., 2018; Ndakidemi et al., 2016). Other anthropogenic pollutants might also contribute to the observed declines in insects, but their impacts are often less well‐studied (Cameron & Sadd, 2020; Feldhaar & Otti, 2020; Sánchez‐Bayo & Wyckhuys, 2019). Airborne particulate matter deriving from traffic or industrial processes has become ubiquitous in the environment (Gieré & Querol, 2010; Zereini & Wiseman, 2010). While the harmful effects on mammals, in particular humans, have been intensively studied, research investigating the impact on insects remains scarce (Kim et al., 2015; Valavanidis et al., 2008). Insects can encounter these pollutants in various ways, for example, by foraging in contaminated areas, consuming contaminated food, or direct deposition on the insect's cuticle (Feldhaar & Otti, 2020; Łukowski et al., 2018; Negri et al., 2015). The airborne particulate matter might enter an insect's body via oral ingestion or the tracheal system (Feldhaar & Otti, 2020; Negri et al., 2015). Social insects might be at an increased risk, as pollutants are transferred to and stored in their nests, which could lead to a higher exposure to conspecifics and the brood (Feldhaar & Otti, 2020; Hladun et al., 2016).

Vehicle brake dust and diesel exhaust particles (DEPs) are major classes of airborne particulate matter deriving from traffic released into the environment (Hamilton & Hartnett, 2013; Harrison et al., 2012; Rönkkö & Timonen, 2019). Brake dust particles contain various metals and phenolic compounds, depending on the brake lining used (Iijima et al., 2007; Thorpe & Harrison, 2008). Exposure of different invertebrate species to such particles showed mixed effects. Particulate matter contamination in soil did not affect colony founding in the ant *Lasius niger* (Seidenath et al., 2021). However, soil‐feeding earthworms (*Eisenia fetida*) showed a strongly increased mortality when exposed to soil spiked with brake dust particles (Holzinger et al., 2022). DEPs have a different composition than brake dust. They are composed of an elemental carbon core with adsorbed organic compounds, such as polycyclic aromatic hydrocarbons (PAHs), and traces of metals and other elements (Greim, 2019; Wichmann, 2007). Exposure to high doses of diesel exhaust particles (1 and 2 g/L) in food over a period of 7 days reduced survival in *Bombus terrestris* workers compared to controls by nearly 50 percent (Hüftlein et al., 2023).

Many classical ecotoxicology approaches focus on the effect of a substance on mortality, growth, or reproduction. However, pollutants can also have more subtle sublethal effects on insects, which may have severe consequences in the long term (Straub et al., 2020). Direct sublethal effects include changes in physiology such as stress reactions or detoxification processes. By interacting with microorganisms inside the insect's body, oral exposure to pollutants may indirectly affect insect health.

Most eukaryotic organisms and their associated microbes form an entity, the so‐called holobiont (Theis et al., 2016; Zilber‐Rosenberg & Rosenberg, 2008). In insects, microorganisms can be found in the digestive tract, the exoskeleton, the hemocoel, or within cells (Douglas, 2015). The insect gut microbiome has a range of functions that include protection from pathogens, detoxification, digestion, and the production of essential nutrients (Engel & Moran, 2013). Social bumblebees (*Bombus* spp.*)* and honeybees (*Apis mellifera*) are model organisms to study gut microbiota as their gut microbiome is rather simple and highly conserved (Engel et al., 2016; Kwong & Moran, 2016; Zhang & Zheng, 2022). A few core bacterial taxa dominate the gut microbiome of bumblebees: *Snodgrassella*, *Gilliamella*, *Schmidhempelia*, Bifidobacteriaceae (*Bifidobacterium and Bombiscardovia*), and two clusters within Lactobacillaceae (Hammer et al., 2021; Koch & Schmid‐Hempel, 2011a; Martinson et al., 2011). While many functions of the bacterial symbionts in bumblebees have been proposed, only very few have been demonstrated in experiments (Hammer et al., 2021; Zhang & Zheng, 2022). The gut microbiome of bumblebees may be important for detoxification as microbiota‐free individuals had lower survival when exposed to toxic concentrations of selenate (Rothman et al., 2019). Moreover, resistance to the common trypanosomatid parasite *Crithidia bombi* is higher in bumblebees with an intact microbiome compared to microbiota‐free individuals (Koch & Schmid‐Hempel, 2011b). When infected with *C. bombi* the outcome varies with host microbiota composition rather than genotype (Koch & Schmid‐Hempel, 2012).

Examining the effects of anthropogenic pollutants, such as airborne particulate matter, on the gut microbiome is an important tool for assessing their risk for insect health (Duperron et al., 2020). Even with a conserved gut microbiome, the relative abundance of core bacteria and the presence of other microorganisms will vary with age, diet, and changing environmental parameters (Koch et al., 2012; Kwong & Moran, 2016). Different pollutants affect the microbial composition of bee guts. In honeybee workers, pesticides or antibiotics change the relative and absolute abundance of core gut microbiota species (DeGrandi‐Hoffman et al., 2017; Motta et al., 2018; Raymann et al., 2017). An array of environmental toxicants, such as cadmium, copper, selenate, and hydrogen peroxide, alter the gut microbiome of *Bombus impatiens* at field‐realistic concentrations (Rothman et al., 2020). These shifts in the microbial community may affect bumblebee health. Intestinal dysbiosis, compositional and functional alteration of the microbiome, is associated with various diseases and health problems in humans and vertebrates (DeGruttola et al., 2016; Levy et al., 2017; Shreiner et al., 2015). In insects, dysbiosis negatively affects reproductive fitness, immunity, and resistance to pathogens (Ami et al., 2010; Daisley et al., 2020; Raymann et al., 2017).

Transcriptome analysis is a sensitive tool to characterize sublethal effects of potentially harmful substances on a molecular and cellular level (Prat & Degli‐Esposti, 2019; Schirmer et al., 2010). Changes in gene expression help to identify biological processes, such as stress responses and detoxification processes, at an early stage. Exposure to different pollutants have been shown to induce changes in gene expression in several insect species. Mosquitos (*Aedes aegypti*) exposed to anthropogenic pollutants (insecticides, PAHs) increased the expression of genes related to detoxification, respiration, and cuticular proteins (David et al., 2010). Fireflies (*Luciola leii*) showed a similar response when exposed to benzo(a)pyrene, a widespread PAH (Zhang et al., 2019). In different bee species, the neonicotinoids imidacloprid, thiamethoxan, and clothianidin induce an upregulation of metabolic, immune, and stress response genes (Aufauvre et al., 2014; Bebane et al., 2019; Christen et al., 2018; Colgan et al., 2019; Gao et al., 2020; Shi et al., 2017). The expression of genes related to detoxification was higher in honeybees (*A. mellifera*) exposed to heavy metals than in controls (Al Naggar et al., 2020; Gizaw et al., 2020; Zhang et al., 2018).

In contrast to pesticides, the effects of other environmental pollutants, such as particulate matter, on gene expression in bees as well as their gut microbiome are largely unclear. To address this knowledge gap, we exposed workers of the buff‐tailed bumblebee *Bombus terrestris* to airborne particulate matter deriving from traffic and investigated changes in the gut microbiome and gene expression. Bumblebees were fed sugar water spiked with sublethal concentrations of brake dust or diesel exhaust particles (DEPs). Adding to this oral exposure, one group of bumblebees was exposed to DEPs via air to enable potential uptake in the tracheal system. We expect changes in the composition of the gut microbial community, as previous research showed changes due to different metals in a closely related *Bombus* species (Rothman et al., 2020). Moreover, we expect changes in the expression of detoxification and metabolic genes, indicating an increased stress level, as the toxic compounds in the particulate matter may interfere with bumblebee physiology.

## 2 METHODS

### 2.1 Bumblebee keeping

Four queenright colonies of *B. terrestris* were ordered from Biobest (Westerlo, Belgium) in March 2021. Colonies were kept in a climate chamber at 26°C and 70% humidity under a constant, inverted 12:12 h light: dark cycle. Colonies were provided with sugar water (50% Apiinvert, Südzucker AG, Mannheim, Germany) and pollen (Imkerpur, Osnabrück, Germany) ad libitum.

### 2.2 Dose selection

The data on airborne particulate matter in terrestrial environments is sparse as it is difficult to quantify and identify the origin. Evidence for high levels of input of airborne particulate matter are often revealed only after it has settled, for example, by analyzing soil samples. Unnaturally high amounts of specific metals could be attributed to external resources such as brake dust (Alsbou & Al‐Khashman, 2018; Peikertova & Filip, 2016). Isotopic analyses of urban soils in Arizona revealed up to 0.54% (w/w) as soot carbon black presumably produced by burning fossil fuels (Hamilton & Hartnett, 2013). While bees are contaminated by airborne particulate matter in the wild, we have no data or modeling on the uptake of these particles (Negri et al., 2015). In previous experiments, chronic oral DEP exposure over 7 days reduced survival of bumblebees when exposed to concentrations of 1 g/L and more (Hüftlein et al., 2023). Oral exposure to brake dust particles reduced survival after 7 days for a concentration of 8 g/L (F. Hüftlein, D. Seidenath, A. Mittereder, T. Hillenbrand, D. Brüggemann, O. Otti, H. Feldhaar, C. Laforsch, M. Schott, unpublished data). For our microbiome and transcriptome experiment we selected sublethal doses of 0.4 g/L that did not affect mortality or fat body weight in previous experiments (F. Hüftlein, D. Seidenath, A. Mittereder, T. Hillenbrand, D. Brüggemann, O. Otti, H. Feldhaar, C. Laforsch, M. Schott, unpublished data). For the flight treatment boxes were contaminated with 1.5 mg of DEP and subsequently single workers released into the boxes. DEP was dispersed by the flight movements of the workers and at this concentration we observed a substantial contamination of the bumblebees on their cuticle in this setup (see below).

### 2.3 Experimental procedure

At the beginning of the experiment, adult workers from the four colonies were randomly assigned to one of six treatments. Control: fed with sugar water only (50% Apiinvert) (*n* = 56); Solvent control: fed with sugar water spiked with 0.02% (v/v) of the emulsifier Tween20 (*n* = 56); Brake dust: fed with sugar water spiked with 0.02% (v/v) of the emulsifier Tween20 and 0.4 g/L brake dust particles (*n* = 56); DEP: fed with sugar water spiked with 0.02% (v/v) of the emulsifier Tween20 and 0.4 g/L diesel exhaust particles (*n* = 56); Flight control: fed with sugar water (50% Apiinvert) and allowed to fly once per day in a plastic box (7 × 7 × 5 cm, EMSA, Emsdetten, Germany) for 3 min (*n* = 24); DEP flight: fed with sugar water (50% Apiinvert) and allowed to fly once per day for 3 min in a plastic box (7 × 7 × 5 cm, EMSA, Emsdetten, Germany) that contained 1.5 (±0.1) mg of diesel exhaust particles (*n* = 24).

The experiment was conducted in a climate chamber at 26°C and 70% humidity under a constant 12:12 h light: dark cycle. Bumblebees were kept in Nicot cages (Nicotplast SAS, Maisod, France) connected to a 12 mL syringe (B. Braun SE, Melsungen, Germany) with the tip cut off, that contained 2 mL of the respective feeding solution (ad libitum). Every day the syringes were replaced with fresh ones to prevent molding or bacterial growth in the food. The exposure lasted for 7 days. At the end of the experiment, the animals were frozen at −20°C.

Within a week after the end of the experiment, we randomly selected twelve (three workers per colony) bumblebees per treatment for transcriptome analysis (*N* = 72). Additionally, for the control, solvent control, brake dust, and DEP treatment, we randomly selected 20 bumblebees (five workers per colony) for microbiome analysis (*N* = 80), respectively.

### 2.4 Generation and collection of diesel exhaust particles (DEPs)

Diesel exhaust particles were collected from a four‐cylinder diesel engine (OM 651, Daimler AG, Stuttgart, Germany) during a repeating cycle of transient and stationary operating points, resembling an inner‐city driving scenario with stop‐and‐go intervals. The engine was operated on a test bench with a water‐cooled eddy‐current brake as previously described in Zöllner (2019). DEP samples were collected by an electrostatic precipitator (OekoTube Inside, Mels‐Plons, Switzerland). A fast response differential mobility particulate spectrometer DMS500 (Combustion, Cambridge, England) was applied to measure submicron particle size distributions of raw exhaust samples. Depending on engine load and speed during the inner‐city cycle, solid particles showed a median diameter between 52.1 ± 1.8 nm and 101.9 ± 1.7 nm. DEP composition was characterized by thermogravimetric analysis (TGA, STA 449 F5 Jupiter, Netzsch‐Gerätebau GmbH, Selb, Germany). A fraction of 72.2% ± 1.1% of the DEP mass was attributed to elemental carbon, 23.2% ± 0.9% w/w to organic fractions, and 4.6% ± 0.7% w/w to inorganic matter. Quantification of PAHs revealed concentrations of 444 ppm for pyrene, 220 ppm for phenanthrene, and 107 ppm for fluoranthene.

The elemental composition of the DEP samples was analyzed by inductively coupled plasma optical emission spectrometry (ICP‐OES, Optima 7300 DV, PerkinElmer Inc., Waltham, United States of America) and interpreted according to Zöllner (2019). It showed fractions of calcium (1.63% w/w), zinc (0.53% w/w), and phosphorus (0.50% w/w) that can be traced back to diesel fuel and lubrication oil. Copper (1.03% w/w), aluminum (0.02% w/w), and iron (0.02% w/w) can be attributed to abrasion of piston rings, cylinder head, and engine block material, respectively. In addition, small amounts of boron (0.13% w/w), magnesium (0.10% w/w), molybdenum (0.03% w/w), natrium (0.02% w/w), and sulfur (0.17% w/w) were found.

### 2.5 Generation of brake dust particles

The brake dust particles provided by the Chair of Ceramic Materials Engineering of the University of Bayreuth are derived from LowMet brake pads (provided by TMD Friction Holdings GmbH, Leverkusen, Germany) that were milled for 3 min in a vibrating cup mill with a tungsten carbide grinding set (Pulverisette 9, Fritsch GmbH, Idar‐Oberstein, Germany). LowMet brake pads are common and representative of passenger cars in Europe and consist of nonferrous metals (25% (w/w)), steel wool (15% (w/w)), petrol coke (12% (w/w)), sulfides (10% (w/w)), aluminum oxide (5% (w/w)), resin (5% (w/w)), graphite (4% (w/w)), mica (4% (w/w)), silicon carbide (3% (w/w)), barite (2% (w/w)), fibers (2% (w/w)), and rubber (1% (w/w)) (Wiaterek, 2012). The particle size distribution of the milled, fine‐grained powder was measured with a laser diffraction particle size analyzer (PSA 1190 LD, Anton Paar GmbH, Ostfildern‐Scharnhausen, Germany). The mean particle size found was 10.19 ± 4.37 μm (D10 = 0.68 μm (10% of all particles being smaller in diameter than this size), D50 = 5.76 μm (median particle size), D90 = 25.87 μm (90% of particles being smaller in diameter than this size)).

### 2.6 Bumblebee gut microbiome analysis

Prior to dissection bumblebees were defrosted and rinsed in 70% ethanol, 90% ethanol, and twice in ultrapure water. We placed each bumblebee on an autoclaved square of aluminum foil (5 × 5 cm) and opened the abdomen with sterilized tweezers and scissors. After carefully separating the midgut and hindgut from the crop and transferring it to an Eppendorf tube, we snap‐froze the gut in liquid nitrogen. All samples were stored at −80°C until further processing.

### 2.7 PCR amplification and sequencing of 16S rDNA fragments

Metagenomic DNA of bumblebee gut samples was purified using the NucleoMag DNA Bacteria kit (Macherey‐Nagel, no. 744310, Düren, Germany) after disruption of samples with 1.4 mm (diam.) ceramic beads (no. P000912‐LYSK0A, Bertin Instruments, Montigny‐le‐Bretonneux, France) in a FastPrep‐24 bead beating device (MPbio, Irvine, USA) following the instructions of the manufacturer. The metagenomic DNA was diluted to a concentration of 5 ng/μL, and 2.5 μL DNA was used to amplify 16S rDNA fragments using primers 515F‐Y (Turner et al., 1999) and 806RB (Apprill et al., 2015) as described in the 16S Metagenomic Sequencing Library Preparation protocol (Part # 15044223 Rev. B, www.illumina.com). Sample libraries were barcoded using the Nextera XT index kit (v2 set A, www.illumina.com), combined in equimolar amounts, and sequenced on Illumina's iSeq‐100 platform using a 293‐cycle single‐end R1 mode. Demultiplexing of reads was performed by the iSeq‐100 local run manager and sample‐specific reads were saved in FastQ format.

### 2.8 Microbiome analysis

Statistical analyses of the microbial data were performed using QIIME2 (Bolyen et al., 2019) and R 4.2.1 (R Core Team, 2022). Forward reads of 16S rDNA fragments (R1 reads) were analyzed using the QIIME2 microbiome analysis package (ver. 2021.11; Bolyen et al., 2019). Unless indicated otherwise, all analysis tools were used as plugins of the QIIME2 package. The respective parameters used along the analysis steps are readily accessible by provenance information in the QIIME2 data files (available as Appendix S1). In brief, the following analysis steps were performed: Demultiplexed reads were trimmed for 16S primer sequences (plugin cutadapt; Martin, 2011), denoised, dereplicated, and chimera‐checked (plugin DADA2; Callahan et al., 2016) resulting in amplified sequence variants (ASVs). Rare ASVs were filtered using the median frequency (=6) of ASVs over all samples. Taxonomic classification of ASVs was performed (plugin feature‐classifier; Bokulich et al., 2018) using the prefitted sklearn‐based taxonomy classifiers based on the SILVA reference database (ver. 138.1; Quast et al., 2013; Yilmaz et al., 2014). ASVs that could not be taxonomically assigned at any taxonomic level (‘unassigned’) as well as samples with less than 3900 reads in total were removed prior to subsequent analysis steps. Alpha diversity metrics, such as Shannon diversity index, Faith's phylogenetic diversity, Pielou's evenness, and observed ASVs, were obtained using the QIIME2's ‘core‐metrics‐phylogenetic’ workflow (plugin diversity), rarefied to 3900 reads per sample. To assess the overall effects of treatment and colony origin on microbial composition we performed permutational multivariate analysis of variance ADONIS from the R package *vegan* (Oksanen et al., 2022) in Qiime2. To find significant differences in α‐diversity we fitted generalized linear mixed models (GLMMs) with treatment as fixed factor and colony as random factor using the function *glmmTMB* from the package *glmmTMB* (Brooks et al., 2017). We checked model assumptions using model diagnostic test plots, that is, qqplot and residual vs. predicted plot from the package *DHARMa* (Hartig, 2022). We then produced statistics with the function *Anova*() from the package *car* (Fox & Weisberg, 2019) to calculate *p*‐values for differences between treatments. For significant treatment effects, we ran pairwise comparisons using Tukey HSD post‐hoc test with Benjamini‐Hochberg correction from the package *multcomp* (Hothorn et al., 2008). Differential abundance of the rarefied data we analyzed using the package *DESeq2* with a negative binomial distribution, a significance level cutoff of FDR < 0.01, replacement of outliers turned off, and cooksCutoff turned off (Love et al., 2014). Compositional differential abundance analysis was performed using Aldex2 (plugin aldex2; Fernandes et al., 2013). Beta diversity of the sparse, compositional microbiome data were calculated using QIIME2's plugin DEICODE, which performs a robust Aitchison PCA (Martino et al., 2019). Significance was tested in a PERMANOVA with 999 permutations followed by pairwise PERMANOVA with Benjamini‐Hochberg (BH) correction for multiple testing (Anderson, 2008). We used the packages *qiime2R* (Bisanz, 2018) and *mia* (Ernst, Shetty, et al., 2022) to import and process the microbiome data in R. Data were arranged using the package *tidyr* (Wickham & Girlich, 2022) and were plotted using the packages *ggplot2* (Wickham, 2016), *ggpubr* (Kassambara, 2020), and *miaViz* (Ernst, Borman, & Lahti, 2022).

### 2.9 Transcriptome analysis of whole bumblebee abdomens

Bumblebees were defrosted and rinsed in 70% ethanol, 90% ethanol, and twice in ultrapure water prior to dissection. The abdomen was cut off with sterile scissors, placed in an Eppendorf tube, and snap‐frozen in liquid nitrogen. All samples were stored at −80°C until further processing.

### 2.10 RNA sequencing

Total RNA was prepared from abdomen samples using the RNeasy Lipid Tissue kit (Qiagen, no. 74804, Hilden, Germany). RNA‐Seq libraries were constructed from 100 ng RNA using the NEBNext Ultra II Directional Library Prep Kit for Illumina (New England Biolabs, no. E7760, Ipswich, USA) in combination with the NEBNext Poly(A) mRNA Magnetic Isolation Module (New England Biolabs, no. E7490, Ipswich, USA). The samples were combined at equimolar amounts and sent out for sequencing on an Illumina device in 150 bp paired‐end mode (Genewiz, Leipzig, Germany). A total of 1.470 million reads, corresponding to an average of 19.5 million reads per sample, were obtained.

### 2.11 Differential expression analysis

RNA‐Seq reads were further analyzed using the OmicsBox bioinformatics platform (v. 2.0.36, www.biobam.com). Unless indicated otherwise, all tools used for differential expression analyses are accessible within the OmicsBox platform. RNA‐Seq reads were preprocessed by Trimmomatic (details see Appendix S1: RNAseq_1_trimmomatic_report) (Bolger et al., 2014) to remove sequencing adapters, low‐quality sequences, and short reads from the dataset. The quality‐trimmed reads were mapped to the *B. terrestris* genome assembly (Bter_1.0, GCA_000214255.1, downloaded from metazoa.ensembl.org) using STAR (Dobin et al., 2013). A gene‐specific count table was created from the mapping files using HTseq (Anders et al., 2015) and differentially expressed genes were identified by edgeR (Robinson et al., 2010), respectively. Functional annotation of the *B. terrestris* genome was based on annotation release v. 102 (available in gff3 format from metazoa.ensembl.org). Since 4975 of the 12,008 genes did not contain any functional annotation, the functional annotation workflow of the OmicsBox platform was used to update the published annotation with additional information. In brief, the coding sequences of unannotated genes were used to extract functional annotations from refseq_protein database (www.ncbi.nlm.nih.gov) and InterProScan (www.ebi.ac.uk). These we then fed into the GO mapping and annotation tools of the pipeline and finally merged to the existing functional annotations. To assess the overall effects of treatment and colony origin on gene expression we performed permutational multivariate analysis of variance ADONIS from the R package vegan (Oksanen et al., 2022) in Qiime2. Gene Set Enrichment Analyses (GSEA; Subramanian et al., 2005) were performed using ranked list of genes (rank = sign(logFC) * −log_10_(*p*‐value); FC: fold change) and gene sets defined by Gene Ontology's functional annotations. For the functional network analysis of enriched GO terms we used ClueGo (v. 2.5.9; Bindea et al., 2009) and CluePedia (v. 1.5.9; Bindea et al., 2013) plugins in Cytoscape (v. 3.9.1; Shannon et al., 2003). We used the packages *ggplot2* (Wickham, 2016), *ggpubr* (Kassambara, 2020), and *pheatmap* (Kolde, 2019) to plot transcriptome data in R 4.2.1 (R Core Team, 2022).

## 3 RESULTS

### 3.1 Effect of pollutants on the bumblebee gut microbiome

**FIGURE A1:**
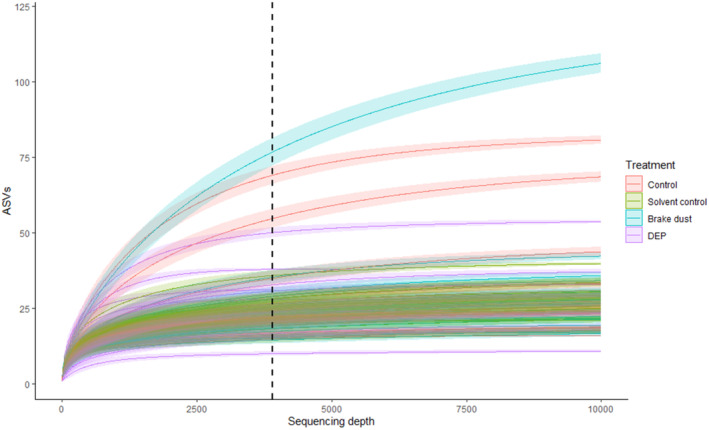
Rarefaction curve of each sample, colored according to their respective treatment. *X*‐Axis is cut off at 10,000 reads. Vertical dashed line indicates sequencing depth of 3900.

Amplicon sequencing of the bacterial 16S rDNA fragments yielded a total of 2,425,928 raw reads. After quality filtering and removal of unassigned sequences, we also removed samples with a sampling depth below 3900 reads (*n* = 7), all from DEP treatment, to ensure adequate sampling depth (13 DEP replicate samples remained in the analysis). In the remaining samples we obtained 1,856,025 16S rDNA gene sequences with a mean of 25,425 reads per sample (*n* = 73), corresponding to 468 amplicon sequence variants (ASVs). Sample‐based rarefaction curves suggest a sufficient sequencing depth for a representative coverage of the microbiome as most of the samples reach a plateau (Figure A1). ADONIS analysis revealed a significant effect of treatment on microbiome composition (*R* ^2^ = 0.423, *p* < .001). There was no significant effect of colony origin (*R* ^2^ = 0.001, *p* = .946) on microbiome composition.

### 3.2 Taxa abundance

**FIGURE 1:**
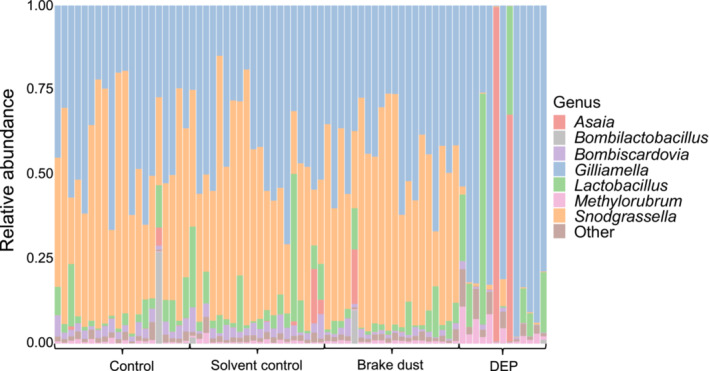
Relative abundance of the most common bacterial genera for each sample. Samples are arranged according to treatment.

On the genus level, the most common bacterial taxa (>1% in at least one treatment) were: *Gilliamella, Snodgrassella, Lactobacillus, Asaia, Bombiscardovia, Methylorubrum*, and *Bombilactobacillus*. The relative abundance of the most common genera for each sample shows a different microbial composition in the DEP treatment compared to the other treatment groups (Figure 1).

**FIGURE 2:**
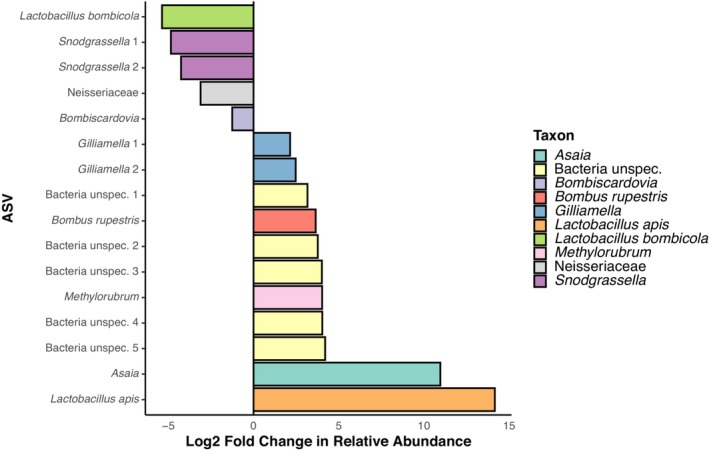
Log_2_ fold change in relative abundance of ASVs in the DEP treatment in comparison to the control. Cutoff for inclusion of ASVs in this plot was FDR (=*p*_adj_) < .01. Colors represent most specific taxonomic label.

While the relative abundance of ASVs did not differ between control, solvent control, and brake dust, DEP treatment had 16 differentially abundant ASVs compared to the control, according to DESeq2 (Figure 2, Table A1). Eleven ASVs had a higher abundance in the DEP treatment than control. Five ASVs had reduced abundance in comparison to the control treatment. A more conservative approach to identify differential abundance is ALDEx2, which revealed five ASVs with significantly altered abundance in the DEP treatment compared to the control: *Snodgrassella* 1 + 2, Neisseriacae, *Lactobacillus bombicola*, and *Bombiscardovia* (Table A2).

### 3.3 α‐diversity of the gut microbiome

**FIGURE 3:**
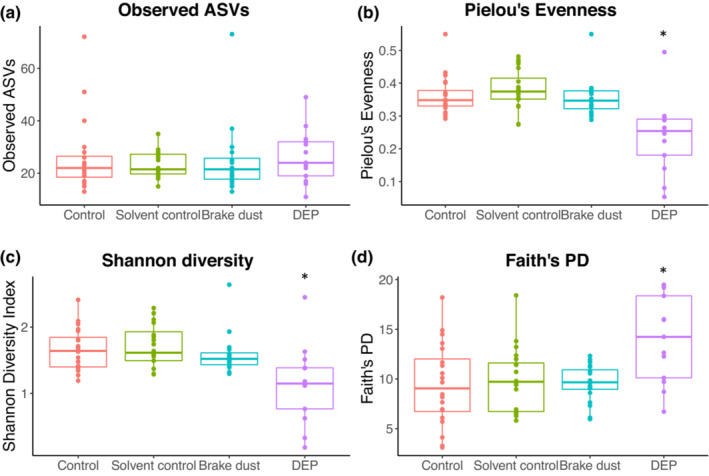
α‐diversity of the bumblebee gut microbiomes for the different treatments. (a) Observed ASVs, (b) Pielou's Evenness, (c) Shannon Diversity, (d) Faith's PD. Asterisks indicate significant differences compared to the other treatments (*p* < .05). Boxplots show median, first, and third quartile. Dots represent individual data points.

The number of observed ASVs did not differ between treatments (GLMM with Gaussian distribution: *χ* ^2^ = 0.918, *df* = 3, *p* = .821; Figure 3a). Pielou's evenness differed between treatments (GLMM with Gaussian distribution: *χ* ^2^ = 42.697, *df* = 3, *p* < .001; Figure 3b). The DEP treatment had a significantly lower evenness than the other treatments (Tukey comparisons with Benjamini‐Hochberg (BH) adjusted *p*‐values: DEP vs. control *p* < .001, DEP vs. solvent control *p* < .001, DEP vs. brake dust *p* < .001; Figure 3b). Shannon diversity differed between treatments (GLMM with Gaussian distribution: *χ* ^2^ = 24.035, *df* = 3, *p* < .001; Figure 3c). The DEP treatment had a significantly lower diversity than the other treatments (Tukey comparisons with BH adjusted *p*‐values: DEP vs. control *p* < .001, DEP vs. solvent control *p* < .001, DEP vs. brake dust *p* < .001; Figure 3c). Faith's PD differed between treatments (GLMM with Gaussian distribution: *χ* ^2^ = 19.062, *df* = 3, *p* < .001; Figure 3d). Faith's PD in the DEP treatment was significantly higher than in the other treatments (Tukey comparisons with BH adjusted *p*‐values: DEP vs. control *p* < .001, DEP vs. solvent control *p* < .001, DEP vs. brake dust *p* < .001; Figure 3d).

### 3.4 β‐diversity of the gut microbiome

**FIGURE 4:**
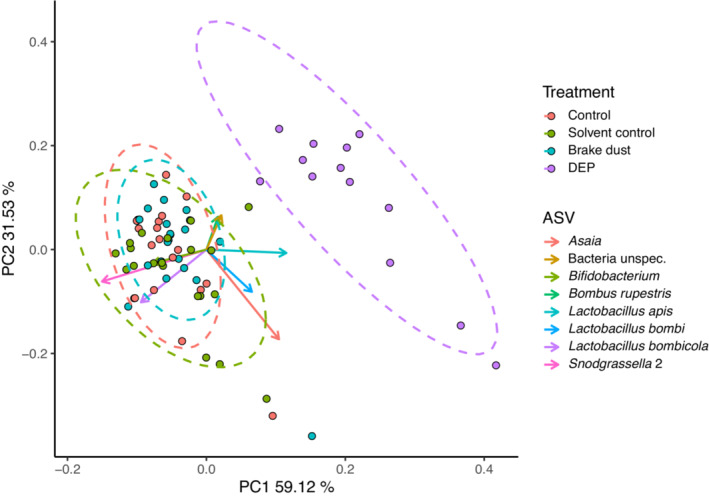
DEICODE distances based on Robust Aitchison Principal Components Analysis. Points represent single samples colored according to treatment. Arrows represent Euclidian distances from the origin and indicate ASVs with strong influence on the principal component axis. Ellipses show 95% confidence interval for multivariate t‐distribution of each treatment. The ASV of the eukaryotic organism *Bombus rupestris* can be explained by a remaining nonspecificity of the used primers (as analyzed by TestPrime, www.arb‐silva.de).

The community composition of the bumblebee gut microbiome differed between treatments indicated by significant differences between the robust Aitchison distances (Overall PERMANOVA pseudo‐*F*_4, 73_ = 16.844, *p* = .001). Microbial community composition of the DEP treatment differed from all other treatments (Pairwise PERMANOVA with BH adjusted *p*‐values; DEP vs. control: pseudo‐*F* = 32.247, *p* = .002; DEP vs. solvent control: pseudo‐*F* = 30.651, *p* = .002; DEP vs. brake dust: pseudo‐*F* = 25.699, *p* = .002). We found no differences between the other treatments (Pairwise PERMANOVA with BH adjusted *p*‐values: *p* > .05) (Figure 4).

### 3.5 Effect of pollutants on bumblebee gene expression

**FIGURE A2:**
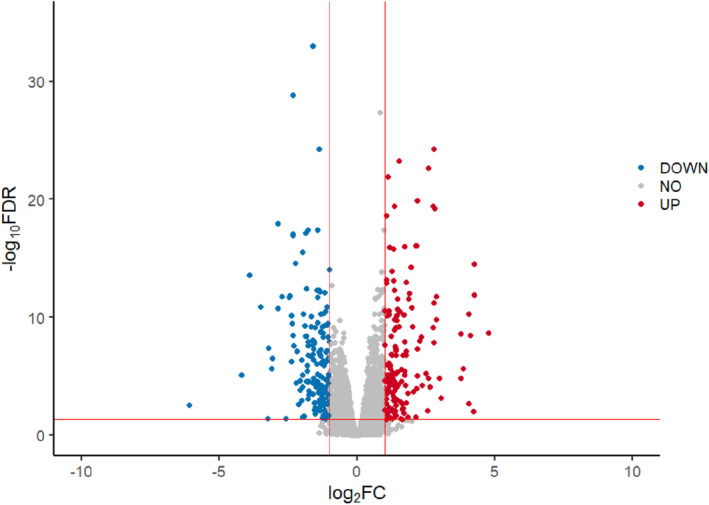
Differential expression of genes in the DEP treatment in comparison to the control. Blue dots represent significantly downregulated genes, red dots represent significantly upregulated genes. The horizontal red line marks a −log_10_(FDR = 0.05). The two vertical red lines mark a log_2_FC of −1 and 1, respectively.

In the transcriptome analysis, we focused only on biologically relevant comparisons of treatments to prevent unnecessary inflation of reported results. We compared control vs. solvent control, control vs. DEP, control vs. brake dust, and flight control vs. DEP flight. The analysis for differently expressed genes (DEGs) revealed differences between our treatments. In total, 324 genes were differentially expressed in the DEP treatment compared to the control (low‐count gene filter settings: CPM Filter = 1, samples reaching CPM Filter = 2). 165 genes were upregulated (LogFC > 1) and 159 genes downregulated (LogFC < −1), respectively (Table A3, Figure A2). In the brake dust treatment only one gene (lipase 3) was differentially expressed (upregulated) in comparison to the control. In the solvent control, there were no differentially expressed genes compared to the control. In the DEP flight treatment, we found no differentially expressed genes in comparison to the flight control. ADONIS analysis revealed a significant effect of treatment on gene expression (*R* ^2^ = 0.279, *p* = .002). There was no significant effect of colony origin (*R* ^2^ = 0.031, *p* = .054) on gene expression.

**FIGURE 5:**
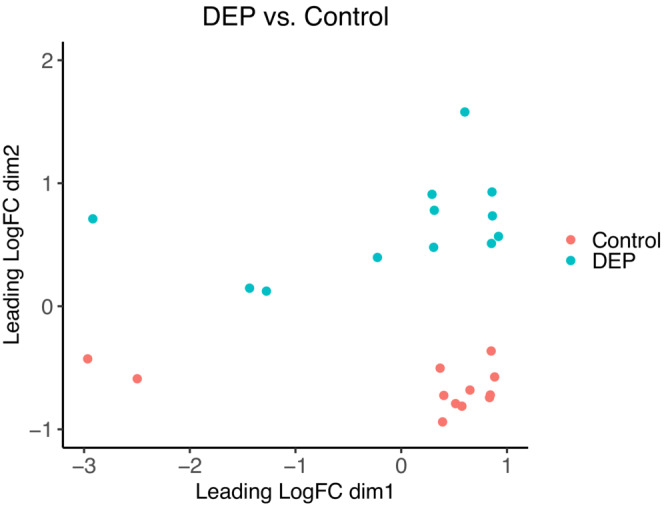
Nonmetric multidimensional scaling plot based on the log_2_ fold changes (FC) between control and DEP treatment. The axes of the nMDS plot represent dimensional reductions of gene expression visualizing the variability of the transcriptional changes for each treatment. Each point represents one sample, colored according to treatment.

**FIGURE 6:**
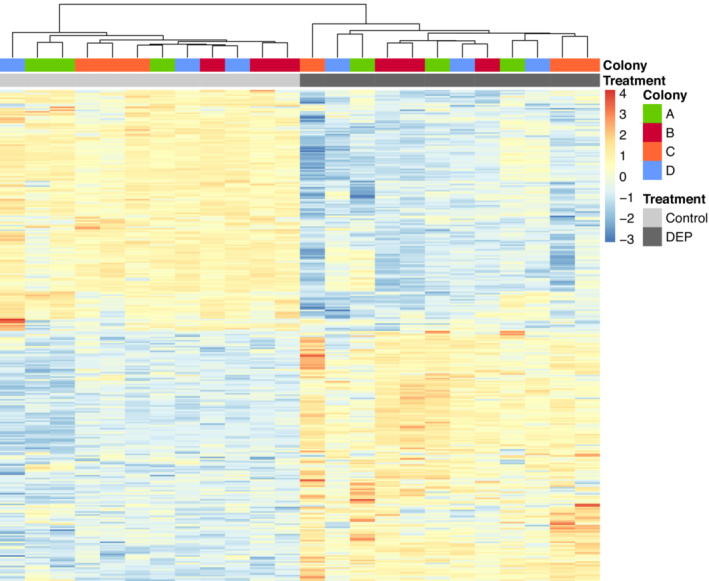
Heatmap showing hierarchical clustering of samples (*x*‐axis) of differentially expressed genes for the control and DEP treatment. The heatmap was obtained using Ward's clustering with the Euclidean distance. The values represent *z*‐scores of log_2_‐transformed CPM (Counts per million reads) expression values.

**FIGURE A3:**
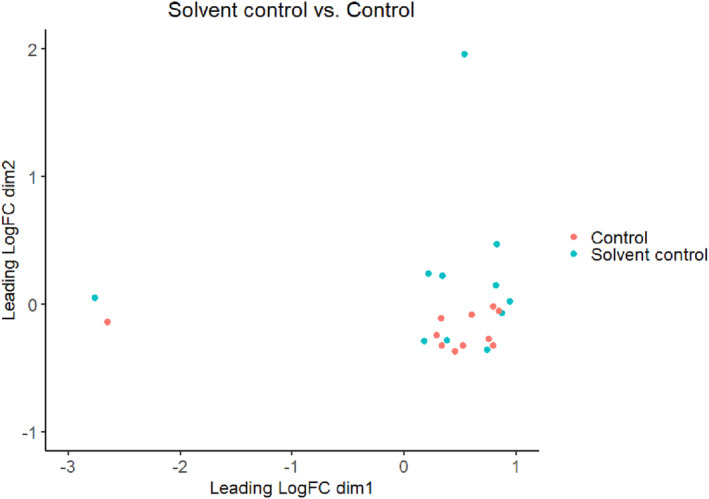
Non‐metric multidimensional scaling plot based on the log_2_ fold changes (FC) between control and solvent control. The axes of the nMDS plot represent dimensional reductions of genes expression visualizing the variability of the transcriptional changes for each treatment. Each point represents one sample, colored according to the respective treatment.

**FIGURE A4:**
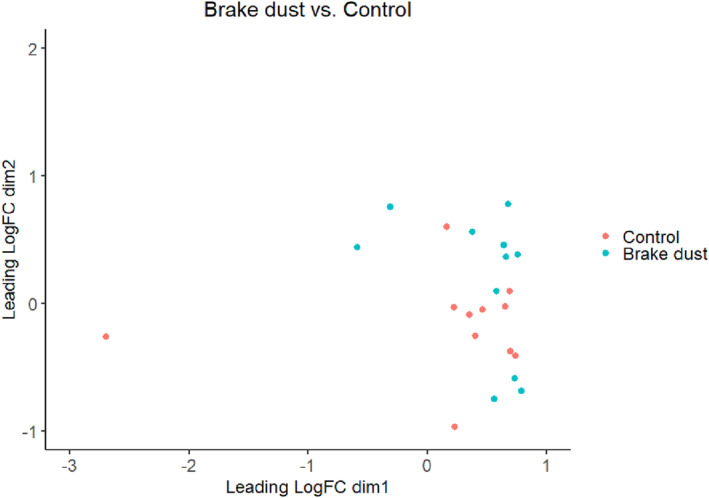
Non‐metric multidimensional scaling plot based on the log_2_ fold changes (FC) between control and brake dust treatment. The axes of the nMDS plot represent dimensional reductions of genes expression visualizing the variability of the transcriptional changes for each treatment. Each point represents one sample, colored according to the respective treatment.

**FIGURE A5:**
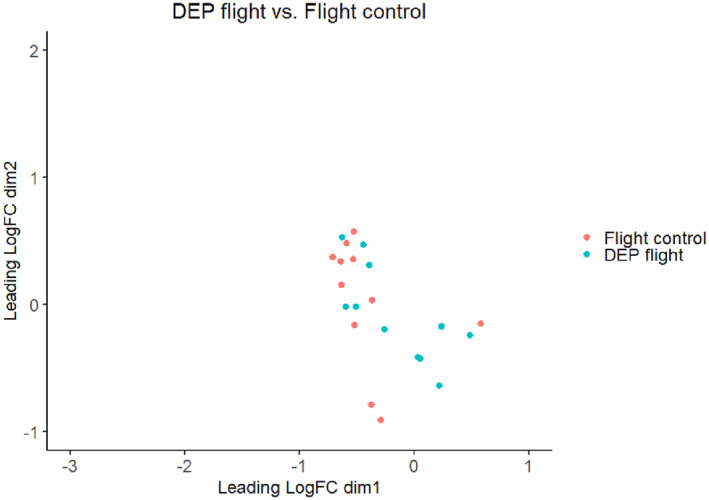
Non‐metric multidimensional scaling plot based on the log_2_ fold changes (FC) between flight control and DEP flight treatment. The axes of the nMDS plot represent dimensional reductions of genes expression visualizing the variability of the transcriptional changes for each treatment. Each point represents one sample, colored according to the respective treatment.

The variation in gene expression of bumblebee workers is clearly distinct between the control and the DEP treatment (Figure 5). The clear separation between the treatments across all samples indicates substantial differences in gene expression of bumblebees when exposed to DEP orally. The reliability of this difference in gene expression is confirmed by a cluster analysis, which shows a definite clustering by treatment rather than by colony (Figure 6). The other treatments are not clearly distinct in a nMDS plot and indicate no differences in gene expression (Figures A3, A4, A5), thus we do not conduct further analyses on these comparisons.

**FIGURE 7:**
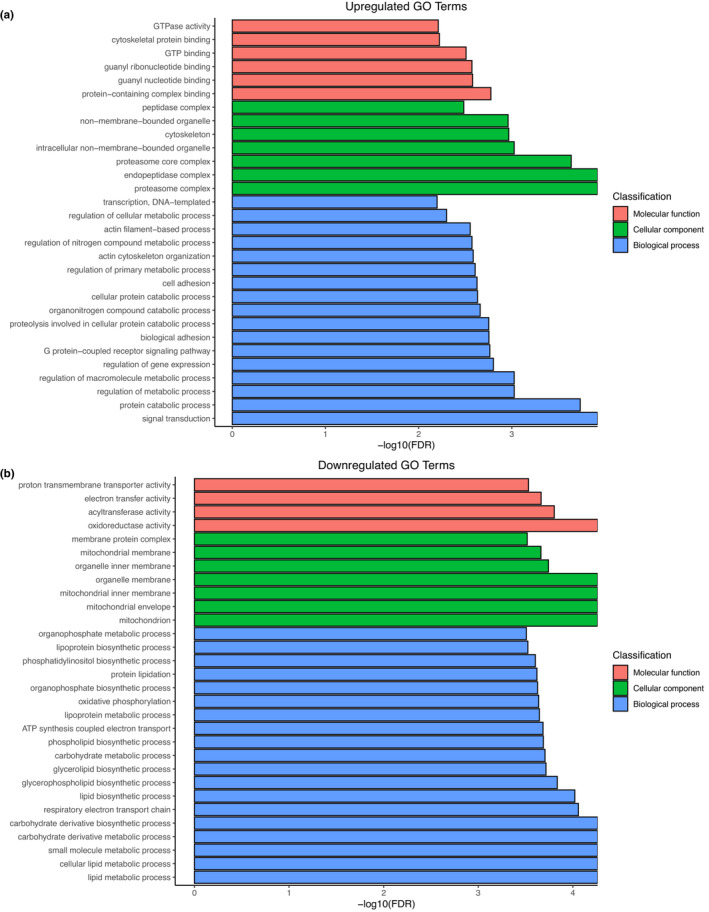
Gene ontology terms of (a) the 30 most significantly upregulated and (b) downregulated genes in the DEP treatment colored by category and sorted by −log_10_FDR.

The 324 differentially expressed genes in the DEP treatment were annotated to gene ontology (GO) terms, which describe gene properties and group each into one of three categories: Cellular component, molecular function, and biological process. We used GO enrichment analysis to find the most over‐ and underrepresented term. The 30 most significantly upregulated GO terms in the DEP treatment include protein‐binding functions, enzyme complexes, and metabolic, especially catabolic, processes (Figure 7a). The 30 most significantly downregulated GO terms in the DEP treatment include transferase activity, mitochondrial and organelle membranes, as well as metabolic, especially biosynthetic, processes (Figure 7b).

**FIGURE A6:**
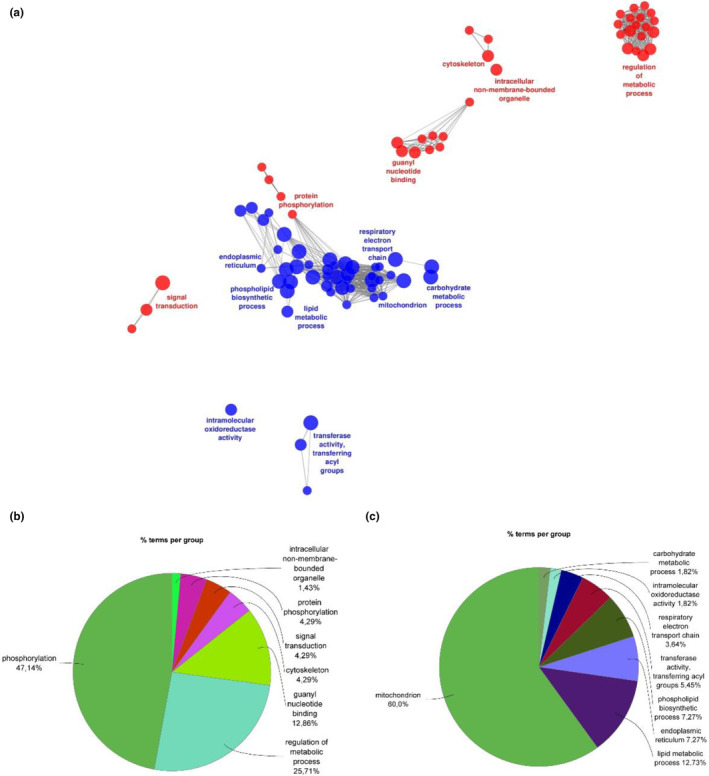
Network analysis of enriched gene terms and functional groups in the DEP treatment based on Kappa‐Score ≥ 0.4 for GOs with FDR ≤ 0.05 using the ClueGo and CluePedia plugins of Cytoscape. (a) Functionally grouped network of upregulated (red) and downregulated (blue) gene ontologies. (b) pie chart with functional groups, including specific terms upregulated in the DEP treatment. (c) pie chart with functional groups, including specific terms downregulated in the DEP treatment. The area covered by each group represents the relative number of GO terms within each group. The most significant term each group is labelled.

The functional network analysis based on 𝜅‐Score ≥ 0.4 for differentially expressed GO terms with FDR ≤0.05 in the DEP treatment shows clustering to specific functional groups (Figure A6a). Upregulated functions are related to phosphorylation, regulation of metabolic process, guanyl nucleotide binding, and signal transduction (Figure A6b). Downregulated functions are related to mitochondria, lipid metabolic processes, the endoplasmic reticulum, and phospholipid biosynthetic processes (Figure A6c).

## 4 DISCUSSION

In this study, we found that oral exposure to diesel exhaust particles (DEPs) changes the gut microbiome and gene expression of bumblebee workers, while DEP exposure via air did not. Brake dust, the second pollutant we tested via oral exposure, did not induce changes in the gut microbiome or gene expression in the bumblebee workers.

While the composition of the microbial gut community in control, solvent control, and brake dust exposure treatment was similar, we detected major shifts in the DEP treatment. This raises several interesting questions: (1) How do DEPs affect the bacteria to induce changes in the gut microbiome composition? (2) Which components in diesel exhaust are responsible for the observed changes? Our hypothesis is that PAHs could be the component of DEP affecting bacteria directly. DEPs contain different PAHs, a class of organic compounds well‐known to be toxic, mutagenic, and genotoxic to various life forms (Patel et al., 2020; Sun et al., 2021). Also, shifts in the microbial gut community due to PAH exposure have been reported in different animals, such as fish, sea cucumbers, or potworms (Enchytraeidae) (DeBofsky et al., 2020, 2021; Ding et al., 2020; Quintanilla‐Mena et al., 2021; Zhao et al., 2019). Therefore, we suspect PAHs to be the leading cause of changes in the bumblebee gut microbiome in our study. However, the large amount of elemental carbon in DEPs, may itself provide another explanation. The DEPs may function like activated carbon with its large surface‐area‐to‐volume ratio and may adsorb microbes that are then discharged by excretion (Naka et al., 2001; Rivera‐Utrilla et al., 2001; Wichmann, 2007). Even though activated carbon has no direct negative impact, constant adsorption and discharge might disrupt the bacterial community resulting in the compositional and quantitative changes similar to those observed in our study.

The bacterium *Snodgrassella*, one of the dominant core bacteria in undisturbed gut microbiomes of bumblebees (Hammer et al., 2021), is nearly absent after the DEP exposure. *Snodgrassella*, together with *Gilliamella*, forms a biofilm coating the inner wall of the ileum (Hammer et al., 2021; Martinson et al., 2012). Both host and symbionts could profit from this biofilm formation as it prevents bacteria from washout and enables the formation of a syntrophic network (Kwong et al., 2014; Powell et al., 2016; Zhang & Zheng, 2022). Additionally, the biofilm could protect the host against gut parasites, such as *C. bombi*, which need to attach to the gut wall to persist (Koch et al., 2019; Näpflin & Schmid‐Hempel, 2018). However, the mutualistic relationship between the microbes seems to be disrupted by DEP exposition, as *Snodgrassella* abundance is extremely diminished. In contrast, *Gilliamella* increases in relative abundance after DEP exposure. This indicates that *Gilliamella* may be able to form a biofilm independently from *Snodgrassella*. A relatively simple explanation for the higher relative abundance of *Gilliamella* might be that the reduction of *Snodgrassella* leaves *Gilliamella* as the only dominant bacterium in the gut, and therefore, *Gilliamella* might thrive better or fill the void. *Snodgrassella* seems especially prone to pollutants, as Rothman et al. (2020) already reported a decrease in its relative abundance after exposure of bees to copper, selenate, or glyphosate. Additionally, we found an unknown bacterium from the family Neisseriaceae, the same family to which also *Snodgrassella* belongs, having a lower relative abundance after DEP exposure. If this is a consistent result, it might indicate a general susceptibility of this family to DEPs.

The higher abundance of *Asaia* in the DEP treatment was driven by two samples, in which *Asaia* dominates the bacterial community with relative abundances of 99% and 67%, respectively. *Asaia* is a flower‐associated acetic acid bacterium, which is commonly found in the gut of members of different insect orders, such as Hemiptera, Diptera, and Hymenoptera (Bassene et al., 2020; Crotti et al., 2009; Kautz et al., 2013). It can dominate the gut microbiome of *Anopheles* mosquitos, which is why it is considered a potential tool in malaria control (Capone et al., 2013; Favia et al., 2008). While there have been reports of *Asaia* in bumblebees, the dominance of *Asaia* in some of the DEP samples is rather uncommon (Bosmans et al., 2018). DEPs might disrupt the natural microbiome community opening the door for opportunistic bacteria such as *Asaia* (Favia et al., 2007). Even though we kept the bumblebees in this experiment indoors throughout their lives, *Asaia* bacteria may derive from pollen fed to the bumblebees before the start of the experiment.

We detected an interesting pattern in the genus *Lactobacillus*, one of the core gut bacteria of bumblebees (Hammer et al., 2021). While the species *L. bombicola*, a bumblebee‐associated bacterium, has a lower abundance after DEP exposure, the abundance of the honeybee‐associated *L. apis* increases. Again, the disruption of the original microbiome caused by DEPs might explain that foreign bacteria can establish themselves in the microbiome. As the pollen fed to the bumblebees before the experiment was collected by honeybees, it could be the source of *L. apis*.

The DEP‐induced changes in the gut microbiome may affect bumblebee health, as core bacteria could prevent infections by parasites. The abundance of *Gilliamella*, *Lactobacillus*, and *Snodgrassella* is negatively correlated with the parasites *Crithidia* and *Nosema*, while noncore bacteria are more abundant in infected bumblebees (Cariveau et al., 2014; Koch et al., 2012; Koch & Schmid‐Hempel, 2012; Mockler et al., 2018). The biofilm formation of *Snodgrassella* and *Gilliamella* may form a physical barrier to the trypanosome *C. bombi*, which needs to attach to the ileum wall to persist (Koch et al., 2019, Näpflin & Schmid‐Hempel, 2018). The disruption of this biofilm and the higher abundance of noncore bacteria, such as *Asaia*, may increase the parasite susceptibility of bumblebees exposed to DEPs.

The transcriptome analysis revealed significant changes in gene expression after oral exposure of bumblebees to a sublethal dose of DEPs. In total, 165 genes were upregulated, and 159 genes were downregulated. GO enrichment analysis and network analysis indicate that these changes could be related to a general stress response against pollutants. While upregulated GO terms involve many metabolic and catabolic processes, downregulated GO terms include metabolic and biosynthetic processes. DEP exposure might deplete stored reserves causing the observed changes as a consequence of higher energetic costs. Changes in metabolism seem to be a typical reaction to pollutants in insects, which seems reasonable as they often interfere with biochemical processes. Transcriptional changes in bumblebees and honeybees exposed to sublethal doses of neonicotinoids are mainly linked to metabolic processes (Bebane et al., 2019; Colgan et al., 2019; Gao et al., 2020; Shi et al., 2017). Exposure to heavy metals or PAHs induces similar changes in spiders, mosquitos, moths, and fireflies (Chen et al., 2021; David et al., 2010; Li et al., 2016; Zhang et al., 2019, 2021). Even though the changes differ in detail, certain processes seem commonly involved in the response to pollutants. Consistent with our findings, exposure to insecticides or PAHs affects mitochondrial functioning, an important part of the insect energy metabolism (Colgan et al., 2019; Zhang et al., 2019, 2021). This supports the idea of increased energy demand caused by pollutants (Beyers et al., 1999; Calow, 1991). We also observed an upregulation of signal transduction in our study, similar to observations in honeybees and fireflies exposed to Imidacloprid and the PAH benzo(a)pyrene, respectively (Gao et al., 2020; Zhang et al., 2019, 2021). Typically, chemical stressors, such as PAHs, insecticides, and heavy metals, affect genes associated with detoxification processes and drug metabolism (Chen et al., 2021; David et al., 2010; Gizaw et al., 2020; Zhang et al., 2019). However, in our study, we did not find any differentially expressed detoxification‐related genes. Possibly the number of PAHs attached to the DEPs was not enough to trigger a reaction that would lead to a measurable increase in detoxification. Overall, the observed changes in gene expression after oral DEP exposure of bumblebees resemble a general stress response to pollutants.

As microbiome and gene expression of bumblebees significantly changed after oral DEP the question arises if and how these systems might affect each other. Metabolic changes may be caused by the DEP‐induced changes in the gut microbiome, which can potentially alter the type and amount of metabolites provided to the host (Douglas, 2018). Moreover, insect immunity might be dependent on gut microbiome. In honeybees the native gut microbiome stimulates immune gene expression, inducing the production of antimicrobial peptides (Kwong et al., 2017). The function and the mechanistic underpinning of this interaction is not entirely clear, but the host might regulate the microbiota in this way. However, host health might also benefit from this interaction by priming the immune system against future infections. Pollutants altering the gut microbiome might thereby jeopardize insect health. This could explain the increased mortality in honeybees with altered gut microbiome due to antibiotic exposure (Raymann et al., 2017).

In contrast to oral exposure, we did not find any effect on gene expression after exposure of bumblebees to DEPs via the air. To cause changes, DEPs need to enter the tracheal system or attach to sensory organs, such as the antennae. The exposure of bumblebees for 3 min per day may not have been enough to affect them. Particles on the antennae may have been removed quickly by cleaning behavior and the spiracles seem to be an effective protective barrier against the uptake of particles into the tracheae (Harrison, 2009; Schönitzer, 1986). Thus, our results should be taken with care because probably only very few particles entered the tracheal system of the bumblebees.

Unlike DEPs, oral exposure to brake dust particles did not affect the gut microbial community or the gene expression of the bumblebees. However, some concerns remain about the experimental procedure. For one, we did not use brake dust from a real braking scenario, but rather artificially milled brake pads. Dust derived from them may have different physicochemical properties. Milled brake dust particles have a much higher mean particle size than DEPs (10 μm vs. 0.01 μm). As we defined treatment concentration per weight, these different physical properties lead to big differences in the particle counts of the treatment solutions, that is, solutions with brake dust contained far fewer particles than those with DEPs. Moreover, large brake dust particles tend to sink to the bottom of the feeding syringes, which might have reduced the particle uptake. While brake dust seems not to affect the bumblebees, further studies are needed to address the indicated limitations of the present study.

Another problem that needs to be addressed is how the doses used in this study relate to field‐realistic concentrations encountered by bumblebees. With the still often vague knowledge of origin and quantity of airborne fine particulate matter present in terrestrial habitats, we know even less about their potential uptake by insects. Contamination of bee products is documented, but there is a need for realistic modeling of encounter rate of insects with airborne particulate matter (Conti & Botrè, 2001). The doses used in this study are presumably higher than those encountered naturally. However, our experimental setup does not include other stressors bees have to face in the wild, such as parasites, limited food availability, or abiotic factors such as drought or heat stress. Bumblebees may be able to compensate for facing one stressor but will eventually be overstrained by multiple stressors.

Taken together, the results from our microbiome and transcriptome analysis indicate potential consequences for insect health, here shown in bumblebees, after oral DEP exposure. Gut dysbiosis may increase the susceptibility of bumblebees to pathogens, while a general stress response may lower available energetic resources. This highlights the potential role of airborne particulate matter such as DEPs as a driver of insect declines.

## AUTHOR CONTRIBUTIONS

**Dimitri Seidenath:** Conceptualization (equal); data curation (equal); formal analysis (equal); investigation (equal); methodology (equal); visualization (lead); writing – original draft (lead). **Alfons R. Weig:** Conceptualization (supporting); data curation (equal); formal analysis (equal); methodology (equal); software (equal); visualization (supporting); writing – original draft (supporting); writing – review and editing (equal). **Andreas Mittereder:** Methodology (equal); resources (equal); writing – review and editing (supporting). **Thomas Hillenbrand:** Methodology (supporting); resources (supporting); supervision (supporting); writing – review and editing (supporting). **Dieter Brüggemann:** Funding acquisition (supporting); project administration (supporting); resources (supporting); supervision (supporting); writing – review and editing (supporting). **Thorsten Opel:** Methodology (supporting); resources (supporting); writing – review and editing (supporting). **Nico Langhof:** Funding acquisition (supporting); methodology (supporting); project administration (supporting); resources (supporting); supervision (supporting); writing – review and editing (supporting). **Marcel Riedl:** Investigation (equal). **Heike Feldhaar:** Conceptualization (equal); funding acquisition (equal); project administration (equal); supervision (equal); writing – original draft (supporting); writing – review and editing (lead). **Oliver Otti:** Conceptualization (equal); formal analysis (supporting); funding acquisition (equal); project administration (equal); supervision (equal); visualization (equal); writing – original draft (supporting); writing – review and editing (lead).

## FUNDING INFORMATION

This project was funded by the Bavarian State Ministry of the Environment and Consumer Protection as part of the project network BayOekotox. The open access publication was funded by the Deutsche Forschungsgemeinschaft (DFG, German Research Foundation) ‐ 491183248. Funded by the Open Access Publishing Fund of the University of Bayreuth.

## Supporting information


Appendix S1.

## Data Availability

The raw data supporting the conclusions of this article will be made available by the authors, without undue reservation. The microbiome and RNA‐Seq sequencing data were deposited at NCBI's Sequence Read Archive (SRA) under Bioproject numbers PRJNA907197 (16S microbiome sequencing) and PRJNA907822 (transcriptome sequencing), respectively.

## APPENDIX A

**TABLE A1:** Differentially abundant ASVs comparing DEP to the control treatment, according to DESeq2 (cutoff: FDR < 0.01).

ASV	Log_2_ fold change	*p*_adj_ (=FDR)	Feature ID
*Lactobacillus bombicola*	−5.372	<.001	ac3366c90455cdc1a4ad414f21215a91
*Snodgrassella* 1	−4.848	<.001	f9dff838e1ab76a58a54df65a2457d5a
*Snodgrassella* 2	−4.256	<.001	8f7166172175c35bbfc8fa4dc5ef58b8
Neisseriaceae	−3.108	<.001	f1ae3848b7e710b5da56f2a447ae0234
*Bombiscardovia*	−1.251	.010	bf7591505d4138d52e3a9c537c958fa1
*Gilliamella* 1	2.146	<.001	36aed5b1dc9b5c1a2844e58f2d34b1f5
*Gilliamella 2*	2.473	<.001	1e232cdf347e2b62b3b1d7347e891797
Bacteria unspec. 1	3.162	.001	6445d5095ad81f1b73aa974a171ebce6
*Bombus rupestris*	3.645	<.001	6d53feb4ee4fac60aba11969e1e5fc01
Bacteria unspec. 2	3.768	.004	101de948d3a66ac329a31fd5f92c00d5
Bacteria unspec. 3	4.008	<.001	7ebb40e08aa315a3ab9ae5fb0b47ae34
*Methylorubrum*	4.025	<.001	92f1720367db58c68a96eceb9feb416a
Bacteria unspec. 4	4.030	<.001	5c70c440562c05d292daf0c5b4694ef4
Bacteria unspec. 5	4.201	<.001	a6ddcd6498df4ed3d6c3e05663f658fb
*Asaia sp*.	10.960	<.001	49d46d00a93443b060707ab2db8ba82d
*Lactobacillus apis*	14.158	<.001	96d14363f547715b65bf7d8ad1d31d17

*Note*: Positive Log_2_ fold changes indicate higher abundance in the DEP treatment.

**TABLE A2:** Differentially abundant ASVs comparing DEP to the control treatment, according to ALDEx2.

ASV	Effect	*p*_adj_	Feature ID
*Snodgrassella* 2	−5.516	<.001	8f7166172175c35bbfc8fa4dc5ef58b8
Neisseriaceae	−2.659	<.001	f1ae3848b7e710b5da56f2a447ae0234
*Lactobacillus bombicola*	−2.393	<.001	ac3366c90455cdc1a4ad414f21215a91
*Snodgrassella* 1	−2.356	<.001	f9dff838e1ab76a58a54df65a2457d5a
*Bombiscardovia*	−2.092	<.001	bf7591505d4138d52e3a9c537c958fa1

*Note*: Negative effect indicates higher abundance in the control. *p*_adj_ = Expected Benjamini‐Hochberg corrected *p* value of Wilcoxon test. Effect = median effect size (diff.btw/max(diff.win)).

**TABLE A3:** Differentially expressed genes in the DEP treatment compared to the control (low‐count gene filter settings: CPM Filter = 1, samples reaching CPM Filter = 2).

Feature	Description	LogFC	LogCPM	FDR
LOC105666082	Protein IWS1 homolog	4.778	−0.174	<0.001
LOC100651567	Protein yellow‐like	4.268	5.560	<0.001
LOC100644846		4.259	5.525	<0.001
LOC100644158		4.239	0.464	0.009
LOC100643093		4.101	2.086	<0.001
LOC105666427	Titin homolog	4.054	−0.636	<0.001
LOC100646940		4.045	−2.033	0.002
LOC110119163	Protein fantom‐like	3.865	−1.153	<0.001
LOC110119507		3.774	−0.774	<0.001
LOC110120240		3.763	−1.264	<0.001
LOC100648995		3.034	2.508	<0.001
LOC100646947	Proline‐rich protein 4	2.981	−0.210	<0.001
LOC100648170	Salivary glue protein Sgs‐3‐like	2.882	4.684	<0.001
LOC110120085	MATH and LRR domain‐containing protein PFE0570w‐like	2.865	0.639	<0.001
LOC100646909	Leucine‐rich repeat protein SHOC‐2‐like isoform X1	2.815	2.525	<0.001
LOC100647974		2.789	9.078	<0.001
LOC100647281	Spore wall protein 2‐like	2.784	0.248	<0.001
LOC100644232	MATH and LRR domain‐containing protein PFE0570w‐like	2.775	4.245	<0.001
LOC100647178		2.754	7.912	<0.001
LOC100645500		2.752	5.742	<0.001
LOC100647176		2.642	−1.240	<0.001
LOC100652307	Mucin‐5AC‐like isoform X3	2.590	3.066	<0.001
LOC100647203	Glycine‐rich cell wall structural protein	2.579	−0.377	<0.001
LOC105666061	Fibrous sheath CABYR‐binding protein‐like	2.551	1.106	0.008
LOC100649104	Electron transfer flavoprotein beta subunit lysine methyltransferase‐like	2.489	−0.274	<0.001
LOC100650993	Hybrid signal transduction histidine kinase L‐like	2.367	−0.563	<0.001
LOC100642564	Proton‐coupled amino acid transporter‐like protein pathetic	2.340	1.911	<0.001
LOC100647041		2.281	4.208	<0.001
LOC100645710	Centrosomal protein of 290 kDa‐like	2.198	4.105	<0.001
LOC100651433		2.187	8.265	<0.001
LOC100644285	Zinc finger protein 100‐like	2.186	0.703	<0.001
LOC100647265		2.163	5.745	<0.001
LOC105666426	Titin homolog	2.151	−0.425	<0.001
LOC100644468	Spore coat protein SP96‐like	2.129	−0.294	0.027
LOC100649167	Coiled‐coil domain‐containing protein 170 isoform X1	2.121	4.034	<0.001
LOC100645585	Uncharacterized protein LOC100645585 isoform X1	2.037	0.733	<0.001
LOC100643561		2.001	6.567	<0.001
LOC100645996		1.988	0.963	<0.001
LOC100652019		1.967	6.469	<0.001
LOC105666709	Uncharacterized protein LOC105666709	1.890	4.064	<0.001
LOC110119744	Uncharacterized protein LOC110119744	1.870	2.413	<0.001
LOC100647550	Cyclin‐dependent kinase inhibitor 1C	1.832	−1.175	0.029
LOC100650340		1.829	−0.412	<0.001
LOC100647929		1.794	−0.292	<0.001
LOC105665898	Uncharacterized protein LOC105665898	1.790	−1.126	0.002
LOC105665941	Uncharacterized protein LOC105665941	1.781	1.785	<0.001
LOC100646677		1.774	9.275	<0.001
LOC110120139	Uncharacterized protein LOC110120139	1.759	0.166	<0.001
LOC100645840		1.739	0.876	<0.001
LOC100647883		1.738	9.769	<0.001
LOC100651423	Cystinosin homolog isoform X1	1.726	7.389	<0.001
LOC110119585	Odorant receptor 49b‐like	1.718	1.471	<0.001
LOC100646153		1.709	2.931	<0.001
LOC105666013	Protein Hook homolog 3‐like	1.687	−0.056	0.013
LOC100645923	Uncharacterized protein LOC100645923 isoform X1	1.657	0.413	<0.001
LOC100649809	Microtubule‐associated protein 10‐like	1.655	−0.359	0.006
LOC100651231		1.645	3.339	0.006
LOC100648688		1.631	4.192	0.046
LOC110119618	Uncharacterized protein LOC110119618	1.630	0.823	0.002
LOC110119338		1.620	0.068	<0.001
LOC100646202		1.617	4.879	<0.001
LOC105666927	Uncharacterized protein LOC105666927	1.576	−0.631	0.041
LOC100651530		1.574	0.299	0.002
LOC100646747		1.562	5.823	<0.001
LOC100648646		1.534	6.323	<0.001
LOC100648300		1.531	7.334	<0.001
LOC110120263	Uncharacterized protein LOC110120263 isoform X2	1.496	4.036	<0.001
LOC100646922		1.473	8.312	<0.001
LOC100648283		1.472	0.806	<0.001
LOC100646009		1.471	9.026	<0.001
LOC100646896		1.449	4.249	<0.001
LOC100649615	Ataxin‐7‐like protein 1	1.440	5.277	<0.001
LOC105666227	LOW QUALITY PROTEIN: uncharacterized protein LOC105666227	1.439	−0.443	0.001
LOC100651732		1.439	2.514	<0.001
LOC100642884		1.436	5.710	<0.001
LOC100642438	Probable WRKY transcription factor protein 1	1.420	3.795	<0.001
VSP		1.409	9.742	<0.001
LOC105666604	Uncharacterized protein LOC105666604	1.404	0.197	0.003
LOC105665882		1.399	2.459	<0.001
LOC100645563		1.399	1.182	<0.001
LOC100646094		1.399	6.502	<0.001
LOC100645979		1.397	−0.803	0.021
LOC105665708	LOW QUALITY PROTEIN: uncharacterized protein LOC105665708	1.394	0.855	0.008
LOC100648236	Uncharacterized protein LOC100648236	1.386	3.095	<0.001
LOC100644599		1.370	4.767	<0.001
LOC100646656	Myb‐like protein X	1.364	1.395	<0.001
LOC100645702		1.361	9.861	<0.001
LOC100652183		1.357	9.345	<0.001
LOC100652258		1.351	9.815	<0.001
LOC100644734		1.344	6.064	<0.001
LOC100642770		1.339	8.595	<0.001
LOC100648102		1.336	9.620	<0.001
LOC100643215		1.335	1.766	0.012
LOC100643695	Vesicular inhibitory amino acid transporter	1.332	0.157	0.001
LOC105666799	Two pore potassium channel protein sup‐9	1.331	0.806	0.033
LOC100648304		1.327	6.114	<0.001
LOC110119815		1.325	3.311	<0.001
LOC100648321	Uncharacterized protein LOC100648321	1.300	2.319	<0.001
LOC100646208	Protein PIH1D3	1.292	0.892	<0.001
LOC100642715		1.288	−0.427	0.009
LOC100647986		1.277	6.942	<0.001
LOC100646384	Pupal cuticle protein G1A‐like	1.275	1.415	<0.001
LOC100645727	Prohormone‐2‐like	1.275	1.824	<0.001
LOC100650276		1.268	2.753	<0.001
LOC100650566		1.264	8.947	<0.001
LOC100649387		1.259	5.304	<0.001
LOC100649836		1.257	3.552	<0.001
LOC100645137		1.252	3.719	<0.001
LOC100648970		1.251	−0.611	0.006
LOC100649938		1.247	6.943	<0.001
LOC100651901		1.242	8.224	<0.001
LOC100646624		1.229	4.294	0.044
LOC100647259	Uncharacterized protein LOC100647259	1.228	5.697	<0.001
LOC100647497		1.213	−0.225	0.008
LOC100649579		1.209	9.855	<0.001
LOC100645676		1.202	7.801	<0.001
LOC100646376		1.195	−0.595	0.033
LOC100649407		1.188	9.355	<0.001
LOC100647950	Alpha‐tocopherol transfer protein‐like	1.184	1.030	0.046
LOC100651491		1.177	2.702	<0.001
LOC100642208	DNA ligase 1‐like isoform X6	1.175	2.630	<0.001
LOC100649496	Uncharacterized protein LOC100649496	1.174	0.985	0.006
LOC100645061	Protein odd‐skipped	1.171	3.722	<0.001
LOC100642957		1.162	6.871	<0.001
LOC105666369		1.162	1.689	0.004
LOC100649739		1.160	7.421	<0.001
LOC100643243		1.153	2.973	<0.001
LOC100648476		1.150	5.734	<0.001
LOC100648653		1.146	2.896	<0.001
LOC100648558		1.143	3.700	<0.001
F2	Uncharacterized abhydrolase domain‐containing protein DDB_G0269086‐like	1.134	−0.529	0.011
LOC100648973	Protein GDAP2 homolog	1.129	9.913	<0.001
LOC105666926	Uncharacterized protein LOC105666926 isoform X2	1.120	2.081	0.0369
LOC100647147		1.115	3.715	<0.001
LOC110119847	Protein lethal(2)essential for life‐like	1.114	4.426	0.039
LOC100645059	SIFamide‐related peptide	1.109	−0.695	0.036
LOC100643782		1.102	0.097	0.003
LOC100644956		1.101	4.921	<0.001
LOC100651177		1.095	2.141	<0.001
LOC100647329		1.091	9.649	<0.001
LOC100646320		1.089	0.916	0.017
LOC100642883		1.081	5.822	<0.001
LOC100651656		1.075	7.739	<0.001
LOC100642484		1.074	−0.079	0.030
LOC100648879		1.072	−0.588	0.043
LOC110119508	Uncharacterized protein LOC110119508	1.072	0.436	<0.001
LOC100642826	Protein FAM151B isoform X2	1.069	9.561	<0.001
LOC100651405	Esterase B1‐like	1.067	0.321	0.003
LOC110119866	Uncharacterized protein LOC110119866	1.065	0.879	0.013
LOC105666040	Uncharacterized protein LOC105666040	1.064	0.860	0.002
LOC100644862		1.052	7.371	<0.001
LOC105666834		1.045	0.993	0.010
LOC100649218	Uncharacterized protein LOC100649218 isoform X2	1.044	1.947	<0.001
LOC100645036		1.040	2.696	<0.001
LOC100649225	Basic proline‐rich protein isoform X1	1.029	−0.635	0.034
LOC100648073		1.026	1.176	0.007
LOC100644397		1.023	1.507	0.015
LOC100644350	Uncharacterized protein LOC100644350	1.018	1.295	0.021
LOC100643873	Prion‐like‐(Q/N‐rich) domain‐bearing protein 25 isoform X2	1.017	8.273	<0.001
LOC100645385		1.016	2.750	<0.001
LOC100647323		1.015	6.130	<0.001
LOC100645062		1.012	−0.628	0.008
LOC100646777		1.009	3.179	0.001
LOC100645806	Growth factor receptor‐bound protein 14 isoform X2	1.004	6.502	<0.001
LOC100644243	Probable salivary secreted peptide	1.002	12.695	<0.001
LOC100649384		−1.008	5.405	<0.001
LOC100650561		−1.017	7.586	<0.001
LOC100643490		−1.018	8.027	<0.001
LOC100649785		−1.019	4.479	<0.001
LOC100647616		−1.020	5.533	0.021
LOC100646229		−1.023	5.296	<0.001
LOC100649475		−1.024	10.228	<0.001
LOC105666138		−1.035	6.859	<0.001
LOC100642963	Histidine‐rich glycoprotein‐like	−1.045	5.154	0.002
LOC100651034		−1.045	6.727	<0.001
LOC100642358		−1.045	3.873	<0.001
LOC100648843		−1.049	8.843	<0.001
LOC100642272		−1.054	8.741	0.021
LOC100631070	Melittin	−1.059	3.766	0.008
LOC100642297	Lysozyme‐like	−1.061	10.682	0.002
LOC100649166		−1.061	4.248	<0.001
LOC100644014		−1.072	6.401	<0.001
LOC100651129	Protein G12	−1.078	10.283	0.043
LOC100645024		−1.091	8.467	<0.001
LOC100644917		−1.091	4.886	<0.001
LOC100647588	Long‐chain fatty acid transport protein 4‐like	−1.096	6.759	<0.001
LOC100644715	Polypeptide N‐acetylgalactosaminyltransferase 2	−1.100	4.682	<0.001
LOC100651969	Uncharacterized protein LOC100651969 isoform X2	−1.100	0.890	0.002
LOC100646207		−1.101	5.682	0.002
LOC105666529	Aquaporin‐11	−1.102	2.117	<0.001
LOC100644235	Uncharacterized protein LOC100644235	−1.107	1.968	<0.001
LOC100646060		−1.114	7.402	<0.001
LOC100648993		−1.115	9.557	<0.001
LOC100648212		−1.117	7.707	<0.001
LOC100646721		−1.135	5.557	<0.001
LOC100646290		−1.146	1.245	0.041
LOC100643349		−1.147	10.562	0.014
LOC100644362		−1.171	5.665	<0.001
LOC100643278	Uncharacterized protein LOC100643278	−1.185	4.474	0.007
LOC100645388		−1.195	5.780	<0.001
LOC100647598		−1.198	3.658	<0.001
LOC100643624		−1.203	8.331	<0.001
LOC100643512		−1.214	8.551	<0.001
LOC100646642		−1.217	2.851	0.003
LOC100642930		−1.218	6.323	<0.001
LOC100646691		−1.219	10.406	<0.001
LOC100649890	Alpha‐tocopherol transfer protein‐like	−1.230	0.789	0.023
LOC100650536		−1.236	6.399	0.002
LOC100651809		−1.236	5.354	<0.001
LOC100649409		−1.241	5.340	<0.001
LOC100645662		−1.243	9.781	<0.001
LOC100649281		−1.253	5.567	<0.001
LOC100648311		−1.261	5.069	<0.001
LOC100646687		−1.265	13.363	<0.001
LOC100643086		−1.268	6.574	<0.001
LOC100646858	Uncharacterized protein LOC100646858	−1.271	1.155	0.035
LOC100650878		−1.281	0.984	0.002
LOC100642446		−1.285	1.803	<0.001
LOC100642488	Ionotropic receptor 75a‐like	−1.292	2.460	<0.001
LOC100646246		−1.311	5.411	<0.001
LOC100649270		−1.312	8.042	<0.001
LOC100648174		−1.312	1.204	0.003
LOC100649872		−1.314	6.120	<0.001
LOC100648029		−1.315	6.794	<0.001
LOC100647832		−1.322	4.922	<0.001
LOC100645755		−1.332	3.458	<0.001
LOC100650947		−1.346	7.668	<0.001
LOC100652063	Trissin	−1.354	1.343	<0.001
LOC100645107		−1.356	8.581	<0.001
LOC100651500	Mid1‐interacting protein 1‐B	−1.357	3.443	<0.001
LOC100645894		−1.359	2.278	<0.001
LOC100645429		−1.360	6.319	<0.001
LOC100650250		−1.364	0.367	0.008
LOC100645461		−1.374	5.010	<0.001
LOC100643020		−1.381	8.281	<0.001
LOC100646701		−1.393	8.105	<0.001
LOC100647539		−1.407	8.027	0.002
LOC100649304		−1.416	6.659	<0.001
LOC100642695		−1.421	−0.396	<0.001
LOC100646080		−1.424	6.440	<0.001
LOC100647261		−1.429	6.854	<0.001
LOC100645568		−1.435	4.554	<0.001
LOC100645839		−1.441	5.714	0.007
LOC100643609		−1.444	3.461	<0.001
LOC100644742		−1.445	4.297	<0.001
LOC100652226		−1.456	6.961	<0.001
LOC100647540		−1.466	5.081	<0.001
LOC100647578		−1.482	7.434	<0.001
LOC100651196		−1.491	6.564	<0.001
LOC100644600		−1.500	6.213	0.006
LOC100652036		−1.510	0.330	0.017
LOC100648169		−1.518	5.991	<0.001
LOC100650111	Uncharacterized protein LOC100650111	−1.531	6.779	0.004
LOC100643779		−1.542	5.867	0.008
LOC100648980		−1.547	4.108	<0.001
LOC100644177		−1.561	2.197	<0.001
LOC100644225		−1.563	8.560	0.002
LOC100644459		−1.572	3.927	<0.001
LOC100644716	Proton‐coupled amino acid transporter‐like protein pathetic	−1.576	6.177	<0.001
LOC100647785		−1.589	9.673	<0.001
LOC100651168	Heterogeneous nuclear ribonucleoprotein A3 homolog 2‐like isoform X2	−1.594	10.511	<0.001
LOC100644772		−1.598	4.675	<0.001
LOC100646078		−1.606	11.649	<0.001
LOC100652210		−1.609	7.475	<0.001
LOC100646491		−1.615	4.672	<0.001
LOC100650628		−1.619	4.326	<0.001
LOC100644921	Proton‐coupled amino acid transporter‐like protein CG1139	−1.621	6.500	<0.001
LOC105667110		−1.668	4.511	<0.001
LOC100645013		−1.669	6.269	<0.001
LOC100646373		−1.674	9.611	<0.001
LOC100649608		−1.681	8.549	<0.001
LOC100645163		−1.751	9.563	<0.001
LOC100652301		−1.753	5.583	<0.001
LOC100649568		−1.763	2.324	<0.001
LOC100652268	Cysteine‐rich venom protein 1‐like isoform X1	−1.773	0.064	<0.001
LOC100648451		−1.778	2.360	<0.001
LOC100649144		−1.781	8.292	<0.001
LOC100646186		−1.783	4.856	<0.001
LOC105666640		−1.791	3.144	<0.001
LOC100647719		−1.794	6.790	<0.001
LOC100647796		−1.795	10.385	<0.001
LOC100646617	Uncharacterized protein LOC100646617	−1.796	8.254	<0.001
LOC100644966	Uncharacterized protein LOC100644966	−1.832	9.266	<0.001
LOC100651268		−1.833	6.843	<0.001
LOC100646752	Uncharacterized protein LOC100646752	−1.837	2.764	<0.001
LOC105666139		−1.875	6.449	<0.001
LOC100646598		−1.877	7.588	<0.001
LOC100650460		−1.900	6.629	<0.001
LOC100643115	Uncharacterized protein LOC100643115	−1.919	5.810	0.023
LOC100644713		−1.959	8.841	<0.001
LOC100644893	Neurotrimin‐like isoform X1	−1.968	5.651	<0.001
LOC100648883		−1.968	8.224	<0.001
LOC100645985	—NA—	−1.985	4.826	0.029
LOC100647222		−1.996	10.755	<0.001
LOC100649178		−2.001	0.465	<0.001
LOC100645831		−2.021	0.541	0.001
LOC100650649		−2.064	7.973	<0.001
LOC100644337	Uncharacterized protein LOC100644337	−2.081	7.848	<0.001
LOC100648482		−2.130	6.922	0.002
LOC100651812		−2.184	7.630	<0.001
LOC100642508		−2.226	2.487	<0.001
LOC105666790		−2.244	3.217	<0.001
LOC100648508	Uncharacterized protein LOC100648508	−2.298	9.712	<0.001
LOC100647241		−2.314	3.493	<0.001
LOC100648563		−2.314	3.255	<0.001
LOC100645349		−2.319	5.686	<0.001
LOC100644867		−2.330	6.433	<0.001
LOC100650704		−2.350	4.803	<0.001
LOC100643391	Zwei Ig domain protein zig‐8	−2.378	4.512	<0.001
LOC105667180	Uncharacterized protein LOC105667180	−2.392	3.648	<0.001
LOC100647739	Cell wall protein RBR3‐like	−2.444	3.621	<0.001
LOC100643622		−2.485	5.346	<0.001
LOC100646104	Endochitinase A1‐like	−2.577	2.397	0.041
LOC100649907		−2.734	6.882	<0.001
LOC100643254	Uncharacterized protein LOC100643254	−2.860	3.945	<0.001
LOC100646690		−2.880	5.792	<0.001
LOC100648425		−3.060	2.570	<0.001
LOC100649744		−3.112	11.820	<0.001
LOC110119840	Lymphocyte expansion molecule‐like	−3.228	−0.248	<0.001
LOC100650436		−3.240	10.056	0.039
LOC100644470		−3.506	6.307	<0.001
LOC100647759		−3.915	10.417	<0.001
LOC100644839		−4.203	3.045	<0.001
LOC100645869	Elastin‐like	−6.097	3.083	0.003

*Note*: Positive log‐fold change (logFC) indicates higher expression in the DEP treatment.
