# Supplementary material for: Diesel exhaust particles alter gut microbiome and gene expression in the bumblebee Bombus terrestris
Source: Ecol Evol. 2023 Jun 21;13(6):e10180. doi: 10.1002/ece3.10180 (PMC10283033; doi:10.1002/ece3.10180)
Supplement: Supplementary file 1 — Appendix S1. [file ECE3-13-e10180-s001.zip › RNAseq_1_trimmomatic_report.pdf]

FASTQ Preprocessing

Input 2: Sequencing Data

A total of 72 libraries have been processed.

Sample Name	Files	Sequencing	Format
Otti-000019	Otti-000019_R1_001.fastq.gz, Otti-000019_R2_001.fastq.gz	Paired-End	FASTQ
Otti-000020	Otti-000020_R1_001.fastq.gz, Otti-000020_R2_001.fastq.gz	Paired-End	FASTQ
Otti-000021	Otti-000021_R1_001.fastq.gz, Otti-000021_R2_001.fastq.gz	Paired-End	FASTQ
Otti-000022	Otti-000022_R1_001.fastq.gz, Otti-000022_R2_001.fastq.gz	Paired-End	FASTQ
Otti-000023	Otti-000023_R1_001.fastq.gz, Otti-000023_R2_001.fastq.gz	Paired-End	FASTQ
Otti-000024	Otti-000024_R1_001.fastq.gz, Otti-000024_R2_001.fastq.gz	Paired-End	FASTQ
Otti-000025	Otti-000025_R1_001.fastq.gz, Otti-000025_R2_001.fastq.gz	Paired-End	FASTQ
Otti-000026	Otti-000026_R1_001.fastq.gz, Otti-000026_R2_001.fastq.gz	Paired-End	FASTQ
Otti-000027	Otti-000027_R1_001.fastq.gz, Otti-000027_R2_001.fastq.gz	Paired-End	FASTQ
Otti-000028	Otti-000028_R1_001.fastq.gz, Otti-000028_R2_001.fastq.gz	Paired-End	FASTQ
Otti-000029	Otti-000029_R1_001.fastq.gz, Otti-000029_R2_001.fastq.gz	Paired-End	FASTQ
Otti-000030	Otti-000030_R1_001.fastq.gz, Otti-000030_R2_001.fastq.gz	Paired-End	FASTQ
Otti-000031	Otti-000031_R1_001.fastq.gz, Otti-000031_R2_001.fastq.gz	Paired-End	FASTQ
Otti-000032	Otti-000032_R1_001.fastq.gz, Otti-000032_R2_001.fastq.gz	Paired-End	FASTQ
Otti-000033	Otti-000033_R1_001.fastq.gz, Otti-000033_R2_001.fastq.gz	Paired-End	FASTQ
Otti-000034	Otti-000034_R1_001.fastq.gz, Otti-000034_R2_001.fastq.gz	Paired-End	FASTQ
Otti-000035	Otti-000035_R1_001.fastq.gz, Otti-000035_R2_001.fastq.gz	Paired-End	FASTQ
Otti-000036	Otti-000036_R1_001.fastq.gz, Otti-000036_R2_001.fastq.gz	Paired-End	FASTQ
Otti-000037	Otti-000037_R1_001.fastq.gz, Otti-000037_R2_001.fastq.gz	Paired-End	FASTQ
Otti-000038	Otti-000038_R1_001.fastq.gz, Otti-000038_R2_001.fastq.gz	Paired-End	FASTQ
Otti-000039	Otti-000039_R1_001.fastq.gz, Otti-000039_R2_001.fastq.gz	Paired-End	FASTQ
Otti-000040	Otti-000040_R1_001.fastq.gz, Otti-000040_R2_001.fastq.gz	Paired-End	FASTQ
Otti-000041	Otti-000041_R1_001.fastq.gz, Otti-000041_R2_001.fastq.gz	Paired-End	FASTQ
Otti-000042	Otti-000042_R1_001.fastq.gz, Otti-000042_R2_001.fastq.gz	Paired-End	FASTQ
Otti-000043	Otti-000043_R1_001.fastq.gz, Otti-000043_R2_001.fastq.gz	Paired-End	FASTQ
Otti-000044	Otti-000044_R1_001.fastq.gz, Otti-000044_R2_001.fastq.gz	Paired-End	FASTQ
Otti-000045	Otti-000045_R1_001.fastq.gz, Otti-000045_R2_001.fastq.gz	Paired-End	FASTQ
Otti-000046	Otti-000046_R1_001.fastq.gz, Otti-000046_R2_001.fastq.gz	Paired-End	FASTQ
Otti-000047	Otti-000047_R1_001.fastq.gz, Otti-000047_R2_001.fastq.gz	Paired-End	FASTQ
Otti-000049	Otti-000049_R1_001.fastq.gz, Otti-000049_R2_001.fastq.gz	Paired-End	FASTQ
Otti-000050	Otti-000050_R1_001.fastq.gz, Otti-000050_R2_001.fastq.gz	Paired-End	FASTQ
Otti-000051	Otti-000051_R1_001.fastq.gz, Otti-000051_R2_001.fastq.gz	Paired-End	FASTQ
Otti-000052	Otti-000052_R1_001.fastq.gz, Otti-000052_R2_001.fastq.gz	Paired-End	FASTQ
Otti-000053	Otti-000053_R1_001.fastq.gz, Otti-000053_R2_001.fastq.gz	Paired-End	FASTQ
Otti-000054	Otti-000054_R1_001.fastq.gz, Otti-000054_R2_001.fastq.gz	Paired-End	FASTQ
Otti-000055	Otti-000055_R1_001.fastq.gz, Otti-000055_R2_001.fastq.gz	Paired-End	FASTQ
Otti-000056	Otti-000056_R1_001.fastq.gz, Otti-000056_R2_001.fastq.gz	Paired-End	FASTQ
Otti-000057	Otti-000057_R1_001.fastq.gz, Otti-000057_R2_001.fastq.gz	Paired-End	FASTQ
Otti-000058	Otti-000058_R1_001.fastq.gz, Otti-000058_R2_001.fastq.gz	Paired-End	FASTQ
Otti-000059	Otti-000059_R1_001.fastq.gz, Otti-000059_R2_001.fastq.gz	Paired-End	FASTQ
Otti-000060	Otti-000060_R1_001.fastq.gz, Otti-000060_R2_001.fastq.gz	Paired-End	FASTQ
Otti-000061	Otti-000061_R1_001.fastq.gz, Otti-000061_R2_001.fastq.gz	Paired-End	FASTQ
Otti-000062	Otti-000062_R1_001.fastq.gz, Otti-000062_R2_001.fastq.gz	Paired-End	FASTQ
Otti-000063	Otti-000063_R1_001.fastq.gz, Otti-000063_R2_001.fastq.gz	Paired-End	FASTQ
Otti-000064	Otti-000064_R1_001.fastq.gz, Otti-000064_R2_001.fastq.gz	Paired-End	FASTQ

Otti-000065	Otti-000065_R1_001.fastq.gz, Otti-000065_R2_001.fastq.gz	Paired-End	FASTQ
Otti-000066	Otti-000066_R1_001.fastq.gz, Otti-000066_R2_001.fastq.gz	Paired-End	FASTQ
Otti-000067	Otti-000067_R1_001.fastq.gz, Otti-000067_R2_001.fastq.gz	Paired-End	FASTQ
Otti-000068	Otti-000068_R1_001.fastq.gz, Otti-000068_R2_001.fastq.gz	Paired-End	FASTQ
Otti-000069	Otti-000069_R1_001.fastq.gz, Otti-000069_R2_001.fastq.gz	Paired-End	FASTQ
Otti-000070	Otti-000070_R1_001.fastq.gz, Otti-000070_R2_001.fastq.gz	Paired-End	FASTQ
Otti-000071	Otti-000071_R1_001.fastq.gz, Otti-000071_R2_001.fastq.gz	Paired-End	FASTQ
Otti-000072	Otti-000072_R1_001.fastq.gz, Otti-000072_R2_001.fastq.gz	Paired-End	FASTQ
Otti-000073	Otti-000073_R1_001.fastq.gz, Otti-000073_R2_001.fastq.gz	Paired-End	FASTQ
Otti-000074	Otti-000074_R1_001.fastq.gz, Otti-000074_R2_001.fastq.gz	Paired-End	FASTQ
Otti-000075	Otti-000075_R1_001.fastq.gz, Otti-000075_R2_001.fastq.gz	Paired-End	FASTQ
Otti-000076	Otti-000076_R1_001.fastq.gz, Otti-000076_R2_001.fastq.gz	Paired-End	FASTQ
Otti-000077	Otti-000077_R1_001.fastq.gz, Otti-000077_R2_001.fastq.gz	Paired-End	FASTQ
Otti-000078	Otti-000078_R1_001.fastq.gz, Otti-000078_R2_001.fastq.gz	Paired-End	FASTQ
Otti-000079	Otti-000079_R1_001.fastq.gz, Otti-000079_R2_001.fastq.gz	Paired-End	FASTQ
Otti-000080	Otti-000080_R1_001.fastq.gz, Otti-000080_R2_001.fastq.gz	Paired-End	FASTQ
Otti-000081	Otti-000081_R1_001.fastq.gz, Otti-000081_R2_001.fastq.gz	Paired-End	FASTQ
Otti-000082	Otti-000082_R1_001.fastq.gz, Otti-000082_R2_001.fastq.gz	Paired-End	FASTQ
Otti-000083	Otti-000083_R1_001.fastq.gz, Otti-000083_R2_001.fastq.gz	Paired-End	FASTQ
Otti-000084	Otti-000084_R1_001.fastq.gz, Otti-000084_R2_001.fastq.gz	Paired-End	FASTQ
Otti-000085	Otti-000085_R1_001.fastq.gz, Otti-000085_R2_001.fastq.gz	Paired-End	FASTQ
Otti-000086	Otti-000086_R1_001.fastq.gz, Otti-000086_R2_001.fastq.gz	Paired-End	FASTQ
Otti-000087	Otti-000087_R1_001.fastq.gz, Otti-000087_R2_001.fastq.gz	Paired-End	FASTQ
Otti-000088	Otti-000088_R1_001.fastq.gz, Otti-000088_R2_001.fastq.gz	Paired-End	FASTQ
Otti-000089	Otti-000089_R1_001.fastq.gz, Otti-000089_R2_001.fastq.gz	Paired-End	FASTQ
Otti-000090	Otti-000090_R1_001.fastq.gz, Otti-000090_R2_001.fastq.gz	Paired-End	FASTQ
Otti-000171	Otti-000171_R1_001.fastq.gz, Otti-000171_R2_001.fastq.gz	Paired-End	FASTQ

Results Overview

Sample	Input Reads	Surviving Reads	Forward Only Surviving Reads	Reverse Only Surviving Reads	Dropped Reads
Otti-000019	19,492,469	18,675,716 / 95.81%	706,210 / 3.62%	22,952 / 0.12%	87,591 / 0.45%
Otti-000020	19,709,759	19,097,037 / 96.89%	455,627 / 2.31%	26,963 / 0.14%	130,132 / 0.66%
Otti-000021	21,932,325	21,393,521 / 97.54%	386,684 / 1.76%	26,551 / 0.12%	125,569 / 0.57%
Otti-000022	18,842,885	18,024,099 / 95.65%	661,226 / 3.51%	23,974 / 0.13%	133,586 / 0.71%
Otti-000023	19,651,599	19,034,329 / 96.86%	414,890 / 2.11%	28,243 / 0.14%	174,137 / 0.89%
Otti-000024	21,251,112	20,600,081 / 96.94%	438,272 / 2.06%	29,146 / 0.14%	183,613 / 0.86%
Otti-000025	23,921,874	22,937,862 / 95.89%	868,770 / 3.63%	26,839 / 0.11%	88,403 / 0.37%
Otti-000026	19,344,863	18,854,689 / 97.47%	342,415 / 1.77%	25,995 / 0.13%	121,764 / 0.63%
Otti-000027	20,269,080	19,725,823 / 97.32%	415,342 / 2.05%	25,585 / 0.13%	102,330 / 0.5%
Otti-000028	17,422,524	16,649,877 / 95.57%	659,826 / 3.79%	21,946 / 0.13%	90,875 / 0.52%
Otti-000029	20,988,812	20,372,696 / 97.06%	474,150 / 2.26%	27,166 / 0.13%	114,800 / 0.55%
Otti-000030	19,585,717	19,132,980 / 97.69%	353,738 / 1.81%	24,130 / 0.12%	74,869 / 0.38%

Otti-000031	18,415,365	17,434,680 / 94.67%	617,760 / 3.35%	30,594 / 0.17%	332,331 / 1.8%
Otti-000032	20,013,910	19,444,796 / 97.16%	423,689 / 2.12%	27,810 / 0.14%	117,615 / 0.59%
Otti-000033	21,944,619	21,347,666 / 97.28%	410,776 / 1.87%	28,285 / 0.13%	157,892 / 0.72%
Otti-000034	17,451,853	16,630,506 / 95.29%	594,050 / 3.4%	26,351 / 0.15%	200,946 / 1.15%
Otti-000035	21,749,531	21,094,252 / 96.99%	538,627 / 2.48%	26,621 / 0.12%	90,031 / 0.41%
Otti-000036	15,725,195	15,092,667 / 95.98%	335,712 / 2.13%	24,825 / 0.16%	271,991 / 1.73%
Otti-000037	17,727,223	16,777,704 / 94.64%	631,435 / 3.56%	27,172 / 0.15%	290,912 / 1.64%
Otti-000038	20,498,902	19,857,387 / 96.87%	403,597 / 1.97%	29,899 / 0.15%	208,019 / 1.01%
Otti-000039	19,545,259	18,965,373 / 97.03%	378,209 / 1.94%	26,635 / 0.14%	175,042 / 0.9%
Otti-000040	18,808,228	17,950,996 / 95.44%	688,973 / 3.66%	23,657 / 0.13%	144,602 / 0.77%
Otti-000041	24,274,699	23,638,551 / 97.38%	537,553 / 2.21%	28,862 / 0.12%	69,733 / 0.29%
Otti-000042	21,411,405	20,832,302 / 97.3%	433,124 / 2.02%	21,467 / 0.1%	124,512 / 0.58%
Otti-000043	25,182,573	24,333,518 / 96.63%	794,337 / 3.15%	23,029 / 0.09%	31,689 / 0.13%
Otti-000044	19,270,218	18,825,360 / 97.69%	360,384 / 1.87%	24,914 / 0.13%	59,560 / 0.31%
Otti-000045	17,807,703	17,426,289 / 97.86%	308,894 / 1.73%	22,195 / 0.12%	50,325 / 0.28%
Otti-000046	20,532,855	19,793,722 / 96.4%	667,943 / 3.25%	23,842 / 0.12%	47,348 / 0.23%
Otti-000047	20,997,491	20,487,382 / 97.57%	424,142 / 2.02%	28,034 / 0.13%	57,933 / 0.28%
Otti-000049	20,610,598	20,164,608 / 97.84%	363,876 / 1.77%	25,079 / 0.12%	57,035 / 0.28%
Otti-000050	19,925,094	19,233,564 / 96.53%	615,638 / 3.09%	22,858 / 0.11%	53,034 / 0.27%
Otti-000051	21,000,061	20,457,455 / 97.42%	421,321 / 2.01%	28,725 / 0.14%	92,560 / 0.44%
Otti-000052	20,084,941	19,634,082 / 97.76%	359,886 / 1.79%	24,378 / 0.12%	66,595 / 0.33%
Otti-000053	19,983,744	19,234,196 / 96.25%	645,983 / 3.23%	25,315 / 0.13%	78,250 / 0.39%
Otti-000054	17,539,692	17,059,035 / 97.26%	378,654 / 2.16%	22,746 / 0.13%	79,257 / 0.45%
Otti-000055	17,795,179	17,385,200 / 97.7%	350,495 / 1.97%	20,764 / 0.12%	38,720 / 0.22%
Otti-000056	20,258,149	19,475,141 / 96.13%	699,699 / 3.45%	26,701 / 0.13%	56,608 / 0.28%
Otti-000057	18,213,662	17,759,648 / 97.51%	340,423 / 1.87%	25,198 / 0.14%	88,393 / 0.49%
Otti-000058	18,921,062	18,505,244 / 97.8%	361,496 / 1.91%	20,430 / 0.11%	33,892 / 0.18%
Otti-000059	18,083,980	17,382,978 / 96.12%	637,879 / 3.53%	21,267 / 0.12%	41,856 / 0.23%
Otti-000060	20,401,679	19,959,610 / 97.83%	386,028 / 1.89%	23,976 / 0.12%	32,065 / 0.16%
Otti-000061	22,281,806	21,803,417 / 97.85%	418,818 / 1.88%	24,544 / 0.11%	35,027 / 0.16%
Otti-000062	21,730,057	20,980,137 / 96.55%	702,231 / 3.23%	21,060 / 0.1%	26,629 / 0.12%

Otti-000063	18,052,708	17,685,465 / 97.97%	321,249 / 1.78%	19,126 / 0.11%	26,868 / 0.15%
Otti-000064	22,202,269	21,725,844 / 97.85%	401,533 / 1.81%	25,484 / 0.11%	49,408 / 0.22%
Otti-000065	18,236,282	17,570,666 / 96.35%	595,307 / 3.26%	22,629 / 0.12%	47,680 / 0.26%
Otti-000066	18,744,249	18,238,246 / 97.3%	434,184 / 2.32%	23,644 / 0.13%	48,175 / 0.26%
Otti-000067	20,194,833	19,723,909 / 97.67%	383,858 / 1.9%	23,940 / 0.12%	63,126 / 0.31%
Otti-000068	18,308,221	17,438,507 / 95.25%	709,174 / 3.87%	23,218 / 0.13%	137,322 / 0.75%
Otti-000069	15,964,448	15,398,846 / 96.46%	362,089 / 2.27%	24,587 / 0.15%	178,926 / 1.12%
Otti-000070	18,094,327	17,589,712 / 97.21%	366,426 / 2.03%	22,183 / 0.12%	116,006 / 0.64%
Otti-000071	18,113,705	17,240,056 / 95.18%	694,632 / 3.83%	22,984 / 0.13%	156,033 / 0.86%
Otti-000072	22,187,475	21,547,900 / 97.12%	526,298 / 2.37%	21,941 / 0.1%	91,336 / 0.41%
Otti-000073	20,332,446	19,577,032 / 96.28%	419,947 / 2.07%	31,060 / 0.15%	304,407 / 1.5%
Otti-000074	17,589,185	16,826,172 / 95.66%	622,231 / 3.54%	19,807 / 0.11%	120,975 / 0.69%
Otti-000075	19,616,332	19,063,122 / 97.18%	467,227 / 2.38%	19,513 / 0.1%	66,470 / 0.34%
Otti-000076	19,610,074	19,030,971 / 97.05%	381,240 / 1.94%	25,948 / 0.13%	171,915 / 0.88%
Otti-000077	17,826,828	17,030,677 / 95.53%	644,578 / 3.62%	23,061 / 0.13%	128,512 / 0.72%
Otti-000078	16,668,671	16,097,469 / 96.57%	351,806 / 2.11%	24,172 / 0.15%	195,224 / 1.17%
Otti-000079	18,297,319	17,659,897 / 96.52%	337,842 / 1.85%	27,049 / 0.15%	272,531 / 1.49%
Otti-000080	17,785,405	16,970,534 / 95.42%	667,152 / 3.75%	22,043 / 0.12%	125,676 / 0.71%
Otti-000081	16,553,912	16,073,423 / 97.1%	366,812 / 2.22%	20,683 / 0.12%	92,994 / 0.56%
Otti-000082	18,918,848	18,413,024 / 97.33%	377,906 / 2%	22,933 / 0.12%	104,985 / 0.55%
Otti-000083	22,013,304	21,127,393 / 95.98%	811,999 / 3.69%	19,108 / 0.09%	54,804 / 0.25%
Otti-000084	19,176,742	18,676,281 / 97.39%	422,088 / 2.2%	21,501 / 0.11%	56,872 / 0.3%
Otti-000085	19,206,458	18,756,570 / 97.66%	366,858 / 1.91%	20,865 / 0.11%	62,165 / 0.32%
Otti-000086	17,600,001	16,796,691 / 95.44%	673,609 / 3.83%	20,260 / 0.12%	109,441 / 0.62%
Otti-000087	17,825,648	17,363,581 / 97.41%	365,280 / 2.05%	21,984 / 0.12%	74,803 / 0.42%
Otti-000088	17,113,142	16,637,511 / 97.22%	394,118 / 2.3%	18,568 / 0.11%	62,945 / 0.37%
Otti-000089	22,590,741	21,771,194 / 96.37%	732,152 / 3.24%	25,300 / 0.11%	62,095 / 0.27%
Otti-000090	20,539,796	20,014,787 / 97.44%	419,826 / 2.04%	23,645 / 0.12%	81,538 / 0.4%
Otti-000171	17,899,292	17,306,958 / 96.69%	341,559 / 1.91%	28,467 / 0.16%	222,308 / 1.24%

Analysis Parameters

Parameter	Value
Upstream Files Pattern	_R1_001
Downstream Files Pattern	_R2_001
Quality Encoding	Autodetection
Remove Adapters	true
Use Adapters From	Default Adapter Sequences
Adapter Sequences	TruSeq3
Seed Mismatches	2
Palindrome Clip Threshold	30
Simple Clip Threshold	15
Minimum Adapter Length	8
Keep Both Reads	true
Trimming	true
Trimming Option	Sliding Window Trimming
Window Size	4
Required Quality	15
Filter By Quality	true
Average Quality	25
Filter By Length	true
Minimum Length	36

References

- Bolger AM., Lohse M. and Usadel B. (2014). Trimmomatic: a flexible trimmer for Illumina sequence data. *Bioinformatics (Oxford, England)*, *30*(15), 2114-20.
- OmicsBox - Bioinformatics made easy. BioBam Bioinformatics (Version 2.0.36). March 3, 2019. www.biobam.com/omicsbox.
