# Supplementary material for: Diesel exhaust particles alter gut microbiome and gene expression in the bumblebee Bombus terrestris
Source: Ecol Evol. 2023 Jun 21;13(6):e10180. doi: 10.1002/ece3.10180 (PMC10283033; doi:10.1002/ece3.10180)
Supplement: Supplementary file 1 — Appendix S1. [file ECE3-13-e10180-s001.zip › RNAseq_2_STAR_mapping_report.pdf]

Read Alignment (STAR) Results

Input 1: Reference Genome Sequences

Bombus_terrestris.Bter_1.0.dna.toplevel

Sequences	Minimum Length	Maximum Length	Average Length	Total Length
5,609	500	18,145,390	44,331	248,654,244

Input 2: FASTQ Files

A total of 72 libraries have been processed.

Sample Name	Files	Sequencing	Format
Otti-000019	Otti-000019_R1_001.fastq.gz, Otti-000019_R2_001.fastq.gz	Paired-End	FASTQ
Otti-000020	Otti-000020_R1_001.fastq.gz, Otti-000020_R2_001.fastq.gz	Paired-End	FASTQ
Otti-000021	Otti-000021_R1_001.fastq.gz, Otti-000021_R2_001.fastq.gz	Paired-End	FASTQ
Otti-000022	Otti-000022_R1_001.fastq.gz, Otti-000022_R2_001.fastq.gz	Paired-End	FASTQ
Otti-000023	Otti-000023_R1_001.fastq.gz, Otti-000023_R2_001.fastq.gz	Paired-End	FASTQ
Otti-000024	Otti-000024_R1_001.fastq.gz, Otti-000024_R2_001.fastq.gz	Paired-End	FASTQ
Otti-000025	Otti-000025_R1_001.fastq.gz, Otti-000025_R2_001.fastq.gz	Paired-End	FASTQ
Otti-000026	Otti-000026_R1_001.fastq.gz, Otti-000026_R2_001.fastq.gz	Paired-End	FASTQ
Otti-000027	Otti-000027_R1_001.fastq.gz, Otti-000027_R2_001.fastq.gz	Paired-End	FASTQ
Otti-000028	Otti-000028_R1_001.fastq.gz, Otti-000028_R2_001.fastq.gz	Paired-End	FASTQ
Otti-000029	Otti-000029_R1_001.fastq.gz, Otti-000029_R2_001.fastq.gz	Paired-End	FASTQ
Otti-000030	Otti-000030_R1_001.fastq.gz, Otti-000030_R2_001.fastq.gz	Paired-End	FASTQ
Otti-000031	Otti-000031_R1_001.fastq.gz, Otti-000031_R2_001.fastq.gz	Paired-End	FASTQ
Otti-000032	Otti-000032_R1_001.fastq.gz, Otti-000032_R2_001.fastq.gz	Paired-End	FASTQ
Otti-000033	Otti-000033_R1_001.fastq.gz, Otti-000033_R2_001.fastq.gz	Paired-End	FASTQ
Otti-000034	Otti-000034_R1_001.fastq.gz, Otti-000034_R2_001.fastq.gz	Paired-End	FASTQ
Otti-000035	Otti-000035_R1_001.fastq.gz, Otti-000035_R2_001.fastq.gz	Paired-End	FASTQ
Otti-000036	Otti-000036_R1_001.fastq.gz, Otti-000036_R2_001.fastq.gz	Paired-End	FASTQ
Otti-000037	Otti-000037_R1_001.fastq.gz, Otti-000037_R2_001.fastq.gz	Paired-End	FASTQ
Otti-000038	Otti-000038_R1_001.fastq.gz, Otti-000038_R2_001.fastq.gz	Paired-End	FASTQ
Otti-000039	Otti-000039_R1_001.fastq.gz, Otti-000039_R2_001.fastq.gz	Paired-End	FASTQ
Otti-000040	Otti-000040_R1_001.fastq.gz, Otti-000040_R2_001.fastq.gz	Paired-End	FASTQ
Otti-000041	Otti-000041_R1_001.fastq.gz, Otti-000041_R2_001.fastq.gz	Paired-End	FASTQ
Otti-000042	Otti-000042_R1_001.fastq.gz, Otti-000042_R2_001.fastq.gz	Paired-End	FASTQ
Otti-000043	Otti-000043_R1_001.fastq.gz, Otti-000043_R2_001.fastq.gz	Paired-End	FASTQ
Otti-000044	Otti-000044_R1_001.fastq.gz, Otti-000044_R2_001.fastq.gz	Paired-End	FASTQ
Otti-000045	Otti-000045_R1_001.fastq.gz, Otti-000045_R2_001.fastq.gz	Paired-End	FASTQ
Otti-000046	Otti-000046_R1_001.fastq.gz, Otti-000046_R2_001.fastq.gz	Paired-End	FASTQ
Otti-000047	Otti-000047_R1_001.fastq.gz, Otti-000047_R2_001.fastq.gz	Paired-End	FASTQ
Otti-000049	Otti-000049_R1_001.fastq.gz, Otti-000049_R2_001.fastq.gz	Paired-End	FASTQ
Otti-000050	Otti-000050_R1_001.fastq.gz, Otti-000050_R2_001.fastq.gz	Paired-End	FASTQ
Otti-000051	Otti-000051_R1_001.fastq.gz, Otti-000051_R2_001.fastq.gz	Paired-End	FASTQ
Otti-000052	Otti-000052_R1_001.fastq.gz, Otti-000052_R2_001.fastq.gz	Paired-End	FASTQ
Otti-000053	Otti-000053_R1_001.fastq.gz, Otti-000053_R2_001.fastq.gz	Paired-End	FASTQ
Otti-000054	Otti-000054_R1_001.fastq.gz, Otti-000054_R2_001.fastq.gz	Paired-End	FASTQ
Otti-000055	Otti-000055_R1_001.fastq.gz, Otti-000055_R2_001.fastq.gz	Paired-End	FASTQ
Otti-000056	Otti-000056_R1_001.fastq.gz, Otti-000056_R2_001.fastq.gz	Paired-End	FASTQ
Otti-000057	Otti-000057_R1_001.fastq.gz, Otti-000057_R2_001.fastq.gz	Paired-End	FASTQ
Otti-000058	Otti-000058_R1_001.fastq.gz, Otti-000058_R2_001.fastq.gz	Paired-End	FASTQ

Otti-000059	Otti-000059_R1_001.fastq.gz, Otti-000059_R2_001.fastq.gz	Paired-End	FASTQ
Otti-000060	Otti-000060_R1_001.fastq.gz, Otti-000060_R2_001.fastq.gz	Paired-End	FASTQ
Otti-000061	Otti-000061_R1_001.fastq.gz, Otti-000061_R2_001.fastq.gz	Paired-End	FASTQ
Otti-000062	Otti-000062_R1_001.fastq.gz, Otti-000062_R2_001.fastq.gz	Paired-End	FASTQ
Otti-000063	Otti-000063_R1_001.fastq.gz, Otti-000063_R2_001.fastq.gz	Paired-End	FASTQ
Otti-000064	Otti-000064_R1_001.fastq.gz, Otti-000064_R2_001.fastq.gz	Paired-End	FASTQ
Otti-000065	Otti-000065_R1_001.fastq.gz, Otti-000065_R2_001.fastq.gz	Paired-End	FASTQ
Otti-000066	Otti-000066_R1_001.fastq.gz, Otti-000066_R2_001.fastq.gz	Paired-End	FASTQ
Otti-000067	Otti-000067_R1_001.fastq.gz, Otti-000067_R2_001.fastq.gz	Paired-End	FASTQ
Otti-000068	Otti-000068_R1_001.fastq.gz, Otti-000068_R2_001.fastq.gz	Paired-End	FASTQ
Otti-000069	Otti-000069_R1_001.fastq.gz, Otti-000069_R2_001.fastq.gz	Paired-End	FASTQ
Otti-000070	Otti-000070_R1_001.fastq.gz, Otti-000070_R2_001.fastq.gz	Paired-End	FASTQ
Otti-000071	Otti-000071_R1_001.fastq.gz, Otti-000071_R2_001.fastq.gz	Paired-End	FASTQ
Otti-000072	Otti-000072_R1_001.fastq.gz, Otti-000072_R2_001.fastq.gz	Paired-End	FASTQ
Otti-000073	Otti-000073_R1_001.fastq.gz, Otti-000073_R2_001.fastq.gz	Paired-End	FASTQ
Otti-000074	Otti-000074_R1_001.fastq.gz, Otti-000074_R2_001.fastq.gz	Paired-End	FASTQ
Otti-000075	Otti-000075_R1_001.fastq.gz, Otti-000075_R2_001.fastq.gz	Paired-End	FASTQ
Otti-000076	Otti-000076_R1_001.fastq.gz, Otti-000076_R2_001.fastq.gz	Paired-End	FASTQ
Otti-000077	Otti-000077_R1_001.fastq.gz, Otti-000077_R2_001.fastq.gz	Paired-End	FASTQ
Otti-000078	Otti-000078_R1_001.fastq.gz, Otti-000078_R2_001.fastq.gz	Paired-End	FASTQ
Otti-000079	Otti-000079_R1_001.fastq.gz, Otti-000079_R2_001.fastq.gz	Paired-End	FASTQ
Otti-000080	Otti-000080_R1_001.fastq.gz, Otti-000080_R2_001.fastq.gz	Paired-End	FASTQ
Otti-000081	Otti-000081_R1_001.fastq.gz, Otti-000081_R2_001.fastq.gz	Paired-End	FASTQ
Otti-000082	Otti-000082_R1_001.fastq.gz, Otti-000082_R2_001.fastq.gz	Paired-End	FASTQ
Otti-000083	Otti-000083_R1_001.fastq.gz, Otti-000083_R2_001.fastq.gz	Paired-End	FASTQ
Otti-000084	Otti-000084_R1_001.fastq.gz, Otti-000084_R2_001.fastq.gz	Paired-End	FASTQ
Otti-000085	Otti-000085_R1_001.fastq.gz, Otti-000085_R2_001.fastq.gz	Paired-End	FASTQ
Otti-000086	Otti-000086_R1_001.fastq.gz, Otti-000086_R2_001.fastq.gz	Paired-End	FASTQ
Otti-000087	Otti-000087_R1_001.fastq.gz, Otti-000087_R2_001.fastq.gz	Paired-End	FASTQ
Otti-000088	Otti-000088_R1_001.fastq.gz, Otti-000088_R2_001.fastq.gz	Paired-End	FASTQ
Otti-000089	Otti-000089_R1_001.fastq.gz, Otti-000089_R2_001.fastq.gz	Paired-End	FASTQ
Otti-000090	Otti-000090_R1_001.fastq.gz, Otti-000090_R2_001.fastq.gz	Paired-End	FASTQ
Otti-000171	Otti-000171_R1_001.fastq.gz, Otti-000171_R2_001.fastq.gz	Paired-End	FASTQ

Results Overview

Unique Reads

Name	Total Reads	Uniquely Mapped Reads	Average Mapped Length	Number of Splices					
				Total	Annot.	GT/AG	GC /AG	AT /AC	Non-canonical
Otti-000019	18,675,716	17,490,661 / 93.655%	280.49	9,813,545	0	9,710,900	57,535	4,027	41,083
Otti-000021	21,393,521	20,037,851 / 93.663%	280.23	10,870,781	0	10,751,165	61,679	4,387	53,550
Otti-000022	18,024,099	16,768,100 / 93.032%	281.57	9,477,415	0	9,369,873	56,966	3,799	46,777
Otti-000020	19,097,037	17,793,877 / 93.176%	281.94	10,218,415	0	10,112,952	59,516	3,949	41,998
Otti-000025	22,937,862	21,283,402 / 92.787%	281.97	11,721,850	0	11,600,491	65,846	4,835	50,678
Otti-000023	19,034,329	17,458,433 / 91.721%	280.35	9,689,257	0	9,587,932	54,146	3,760	43,419
Otti-000024	20,600,081	19,152,937 / 92.975%	282.38	10,553,819	0	10,435,245	64,203	3,746	50,625

Otti-000026	18,854,689	17,788,765 / 94.347%	278.84	9,963,408	0	9,860,886	54,705	4,011	43,806
Otti-000027	19,725,823	18,133,596 / 91.928%	281.33	9,768,961	0	9,664,901	52,736	3,911	47,413
Otti-000028	16,649,877	15,743,580 / 94.557%	280.09	8,994,680	0	8,899,945	54,299	3,750	36,686
Otti-000029	20,372,696	19,036,993 / 93.444%	281.56	10,530,600	0	10,419,899	61,438	4,507	44,756
Otti-000030	19,132,980	17,883,992 / 93.472%	279.19	9,688,523	0	9,582,891	58,490	3,798	43,344
Otti-000031	17,434,680	16,257,769 / 93.25%	277.3	8,796,189	0	8,705,463	48,184	3,846	38,696
Otti-000032	19,444,796	18,094,060 / 93.053%	279.6	9,519,481	0	9,420,758	52,809	3,880	42,034
Otti-000033	21,347,666	19,850,506 / 92.987%	281.03	10,505,652	0	10,397,676	57,331	4,154	46,491
Otti-000034	16,630,506	15,292,148 / 91.952%	278.87	8,094,316	0	8,011,844	43,554	3,175	35,743
Otti-000035	21,094,252	19,544,099 / 92.651%	281.92	10,477,753	0	10,368,958	56,463	4,223	48,109
Otti-000036	15,092,667	13,945,387 / 92.398%	279.44	7,220,137	0	7,146,906	38,026	3,033	32,172
Otti-000037	16,777,704	15,503,936 / 92.408%	279.35	8,220,392	0	8,138,444	41,890	3,621	36,437
Otti-000038	19,857,387	18,354,667 / 92.432%	276.83	9,187,951	0	9,095,085	45,621	4,223	43,022
Otti-000039	18,965,373	17,785,969 / 93.781%	280.14	9,705,761	0	9,607,917	50,842	4,237	42,765
Otti-000040	17,950,996	16,682,774 / 92.935%	277.52	9,364,939	0	9,268,048	56,378	3,485	37,028
Otti-000041	23,638,551	22,297,041 / 94.325%	280.18	12,239,599	0	12,117,887	69,233	5,120	47,359
Otti-000044	18,825,360	17,621,574 / 93.606%	277.39	9,731,532	0	9,631,208	57,641	3,776	38,907
Otti-000045	17,426,289	16,105,950 / 92.423%	280.61	9,003,050	0	8,906,996	52,038	3,572	40,444
Otti-000046	19,793,722	18,282,650 / 92.366%	281.05	10,354,169	0	10,238,858	62,576	3,824	48,911
Otti-000047	20,487,382	19,007,033 / 92.774%	279.04	10,194,020	0	10,092,504	56,415	3,863	41,238
Otti-000049	20,164,608	18,290,737 / 90.707%	279.49	9,520,837	0	9,421,336	51,526	3,791	44,184
Otti-000050	19,233,564	17,836,814 / 92.738%	280.35	9,944,338	0	9,837,324	59,440	3,870	43,704
Otti-000052	19,634,082	18,114,519 / 92.261%	281.95	9,809,480	0	9,712,797	54,029	4,049	38,605
Otti-000051	20,457,455	18,463,739 / 90.254%	279.85	9,789,173	0	9,687,903	50,494	3,919	46,857
Otti-000053	19,234,196	17,667,276 / 91.853%	278.91	9,342,830	0	9,244,428	51,448	3,618	43,336
Otti-000054	17,059,035	15,593,422 / 91.409%	280.32	8,143,000	0	8,059,802	43,550	3,258	36,390
Otti-000055	17,385,200	15,842,866 / 91.128%	282.95	8,817,132	0	8,721,464	49,285	3,156	43,227
Otti-000056	19,475,141	17,874,353 / 91.78%	279.91	8,915,121	0	8,818,040	48,522	4,018	44,541
Otti-000057	17,759,648	16,331,985 / 91.961%	279.1	8,552,550	0	8,468,136	44,200	3,389	36,825
Otti-000059	17,382,978	16,049,844 / 92.331%	279.7	9,133,265	0	9,037,872	52,641	3,257	39,495
Otti-000060	19,959,610	18,742,209 / 93.901%	280.1	10,360,045	0	10,253,465	59,838	3,849	42,893

Otti-000058	18,505,244	14,418,824 / 77.918%	280.66	8,247,664	0	8,154,741	49,600	3,042	40,281
Otti-000062	20,980,137	12,545,609 / 59.798%	280.58	7,291,899	0	7,209,681	46,807	2,447	32,964
Otti-000042	20,832,302	4,539,039 / 21.788%	279.29	2,529,629	0	2,503,432	14,405	1,215	10,577
Otti-000064	21,725,844	20,396,772 / 93.883%	280.88	11,638,327	0	11,525,074	65,437	4,376	43,440
Otti-000061	21,803,417	12,362,865 / 56.702%	281.69	6,946,770	0	6,877,641	40,231	2,635	26,263
Otti-000065	17,570,666	16,344,111 / 93.019%	280.03	9,043,227	0	8,947,213	53,953	3,606	38,455
Otti-000066	18,238,246	17,111,292 / 93.821%	283.22	9,349,344	0	9,255,632	52,769	3,368	37,575
Otti-000067	19,723,909	18,234,954 / 92.451%	281.03	10,016,949	0	9,909,797	60,616	3,688	42,848
Otti-000069	15,398,846	14,298,627 / 92.855%	279.57	7,436,955	0	7,362,105	39,049	3,184	32,617
Otti-000070	17,589,712	16,363,054 / 93.026%	282.25	8,794,880	0	8,704,439	49,105	3,340	37,996
Otti-000068	17,438,507	15,435,811 / 88.516%	281.2	8,422,870	0	8,338,950	44,574	3,199	36,147
Otti-000071	17,240,056	15,779,096 / 91.526%	281.28	8,560,609	0	8,472,964	46,513	3,162	37,970
Otti-000043	24,333,518	4,325,591 / 17.776%	275.94	2,421,708	0	2,397,000	14,623	764	9,321
Otti-000063	17,685,465	7,239,706 / 40.936%	278.32	3,947,511	0	3,904,492	23,619	1,602	17,798
Otti-000073	19,577,032	17,926,048 / 91.567%	279.78	9,548,512	0	9,436,944	53,934	3,977	53,657
Otti-000076	19,030,971	17,812,611 / 93.598%	281.03	9,710,011	0	9,606,597	55,770	4,460	43,184
Otti-000077	17,030,677	15,911,516 / 93.429%	278.44	9,029,856	0	8,936,972	52,791	3,384	36,709
Otti-000078	16,097,469	14,911,611 / 92.633%	278.75	8,381,741	0	8,296,789	46,886	3,091	34,975
Otti-000079	17,659,897	16,598,210 / 93.988%	279.13	9,257,418	0	9,159,129	50,189	3,385	44,715
Otti-000081	16,073,423	14,993,878 / 93.284%	283.49	8,414,341	0	8,327,342	48,765	3,072	35,162
Otti-000082	18,413,024	17,064,541 / 92.676%	283.1	9,769,442	0	9,666,070	58,122	3,524	41,726
Otti-000080	16,970,534	14,309,837 / 84.322%	282.1	7,822,101	0	7,737,379	43,590	2,901	38,231
Otti-000074	16,826,172	8,885,116 / 52.805%	278.87	4,563,687	0	4,511,219	25,151	1,735	25,582
Otti-000085	18,756,570	17,489,909 / 93.247%	284.47	9,859,540	0	9,759,005	57,543	3,515	39,477
Otti-000084	18,676,281	13,680,609 / 73.251%	283.6	7,787,946	0	7,705,473	45,218	3,041	34,214
Otti-000086	16,796,691	15,689,030 / 93.405%	282.93	8,778,615	0	8,682,055	54,652	3,364	38,544
Otti-000087	17,363,581	16,308,926 / 93.926%	280.79	8,595,883	0	8,504,801	47,120	3,771	40,191
Otti-000088	16,637,511	15,560,343 / 93.526%	282.23	8,722,668	0	8,631,360	51,027	3,478	36,803
Otti-000089	21,771,194	20,514,042 / 94.226%	282.41	11,427,731	0	11,302,218	64,131	5,337	56,045
Otti-000090	20,014,787	18,430,598 / 92.085%	282.04	10,744,852	0	10,630,882	58,845	3,930	51,195
Otti-000075	19,063,122	6,116,089 / 32.083%	281.62	3,319,745	0	3,285,772	17,919	1,368	14,686

Otti-000171	17,306,958	15,845,889 / 91.558%	278.45	7,489,967	0	7,405,706	40,995	3,921	39,345
Otti-000072	21,547,900	3,606,402 / 16.737%	281.27	1,809,982	0	1,790,979	9,204	918	8,881
Otti-000083	21,127,393	4,766,337 / 22.56%	282.34	2,642,269	0	2,616,362	14,146	982	10,779

Name	Mismatch Rate per Base (%)	Deletions		Insertions	
		Rate per Base (%)	Average Length	Rate per Base (%)	Average Length
Otti-000019	0.35	0.02	1.8	0.03	1.81
Otti-000021	0.34	0.02	1.9	0.03	1.8
Otti-000022	0.36	0.02	1.88	0.03	1.8
Otti-000020	0.38	0.02	1.84	0.03	1.8
Otti-000025	0.36	0.02	1.86	0.03	1.8
Otti-000023	0.41	0.02	1.88	0.03	1.83
Otti-000024	0.36	0.02	1.91	0.03	1.81
Otti-000026	0.37	0.02	1.8	0.03	1.81
Otti-000027	0.37	0.02	1.89	0.03	1.83
Otti-000028	0.37	0.02	1.82	0.03	1.81
Otti-000029	0.37	0.02	1.82	0.03	1.84
Otti-000030	0.35	0.02	1.83	0.03	1.87
Otti-000031	0.37	0.02	1.83	0.03	1.81
Otti-000032	0.39	0.02	1.77	0.03	1.82
Otti-000033	0.36	0.02	1.84	0.03	1.84
Otti-000034	0.39	0.02	1.79	0.03	1.83
Otti-000035	0.4	0.02	1.8	0.03	1.85
Otti-000036	0.38	0.02	1.8	0.03	1.83
Otti-000037	0.39	0.02	1.8	0.03	1.86
Otti-000038	0.42	0.02	1.73	0.03	1.88
Otti-000039	0.37	0.02	1.8	0.03	1.82
Otti-000040	0.36	0.02	1.8	0.03	1.78
Otti-000041	0.35	0.02	1.9	0.03	1.78
Otti-000044	0.38	0.02	1.82	0.03	1.8
Otti-000045	0.36	0.02	1.87	0.03	1.79
Otti-000046	0.38	0.02	1.83	0.03	1.8

Otti-000047	0.37	0.02	1.86	0.03	1.8
Otti-000049	0.39	0.02	1.71	0.03	1.87
Otti-000050	0.36	0.02	1.81	0.03	1.83
Otti-000052	0.35	0.02	1.84	0.03	1.8
Otti-000051	0.41	0.02	1.84	0.03	1.84
Otti-000053	0.38	0.02	1.79	0.03	1.84
Otti-000054	0.41	0.02	1.79	0.03	1.84
Otti-000055	0.37	0.02	1.85	0.03	1.83
Otti-000056	0.4	0.02	1.82	0.04	1.87
Otti-000057	0.4	0.02	1.78	0.03	1.84
Otti-000059	0.35	0.02	1.87	0.03	1.76
Otti-000060	0.36	0.02	1.93	0.03	1.76
Otti-000058	0.35	0.02	1.95	0.03	1.8
Otti-000062	0.34	0.02	2.01	0.03	1.83
Otti-000042	0.38	0.02	1.82	0.03	1.81
Otti-000064	0.35	0.02	1.8	0.03	1.82
Otti-000061	0.34	0.02	1.83	0.03	1.82
Otti-000065	0.36	0.02	1.84	0.03	1.78
Otti-000066	0.38	0.02	1.81	0.03	1.82
Otti-000067	0.34	0.02	1.84	0.03	1.82
Otti-000069	0.4	0.02	1.83	0.03	1.81
Otti-000070	0.36	0.02	1.89	0.03	1.86
Otti-000068	0.38	0.02	1.82	0.03	1.84
Otti-000071	0.38	0.02	1.87	0.03	1.88
Otti-000043	0.35	0.02	1.83	0.03	1.78
Otti-000063	0.35	0.02	1.94	0.03	1.79
Otti-000073	0.37	0.02	1.89	0.03	1.85
Otti-000076	0.33	0.02	1.88	0.03	1.78
Otti-000077	0.35	0.02	1.92	0.03	1.81
Otti-000078	0.38	0.02	1.85	0.03	1.8
Otti-000079	0.36	0.02	1.9	0.03	1.8

Otti-000081	0.38	0.02	1.92	0.03	1.81
Otti-000082	0.35	0.02	1.91	0.03	1.81
Otti-000080	0.36	0.02	1.88	0.03	1.78
Otti-000074	0.38	0.02	1.9	0.03	1.82
Otti-000085	0.35	0.02	1.92	0.03	1.82
Otti-000084	0.37	0.02	1.89	0.03	1.81
Otti-000086	0.36	0.02	1.85	0.03	1.81
Otti-000087	0.36	0.02	1.91	0.03	1.81
Otti-000088	0.35	0.02	1.87	0.03	1.79
Otti-000089	0.32	0.02	1.93	0.03	1.8
Otti-000090	0.38	0.02	1.89	0.03	1.77
Otti-000075	0.39	0.02	1.87	0.03	1.82
Otti-000171	0.41	0.03	1.74	0.04	1.88
Otti-000072	0.43	0.03	1.77	0.04	1.85
Otti-000083	0.36	0.02	1.83	0.03	1.78

Multi-mapping Reads

Name	Reads Mapped to Multiple Loci	Reads Mapped to too Many Loci
Otti-000019	329,114 / 1.762%	215 / 0.001%
Otti-000021	345,617 / 1.616%	250 / 0.001%
Otti-000022	326,838 / 1.813%	237 / 0.001%
Otti-000020	346,839 / 1.816%	232 / 0.001%
Otti-000025	432,426 / 1.885%	280 / 0.001%
Otti-000023	293,708 / 1.543%	280 / 0.001%
Otti-000024	365,907 / 1.776%	301 / 0.001%
Otti-000026	276,730 / 1.468%	294 / 0.002%
Otti-000027	403,145 / 2.044%	273 / 0.001%
Otti-000028	215,630 / 1.295%	250 / 0.002%
Otti-000029	374,825 / 1.84%	249 / 0.001%
Otti-000030	359,624 / 1.88%	234 / 0.001%
Otti-000031	284,324 / 1.631%	245 / 0.001%
Otti-000032	378,165 / 1.945%	282 / 0.001%
Otti-000033	461,403 / 2.161%	350 / 0.002%
Otti-000034	322,002 / 1.936%	260 / 0.002%
Otti-000035	440,102 / 2.086%	316 / 0.001%
Otti-000036	332,813 / 2.205%	272 / 0.002%
Otti-000037	312,791 / 1.864%	265 / 0.002%
Otti-000038	419,024 / 2.11%	346 / 0.002%
Otti-000039	282,171 / 1.488%	333 / 0.002%
Otti-000040	324,168 / 1.806%	240 / 0.001%
Otti-000041	384,157 / 1.625%	272 / 0.001%
Otti-000044	273,783 / 1.454%	249 / 0.001%

Otti-000045	351,377 / 2.016%	267 / 0.002%
Otti-000046	294,628 / 1.488%	264 / 0.001%
Otti-000047	421,342 / 2.057%	240 / 0.001%
Otti-000049	408,900 / 2.028%	289 / 0.001%
Otti-000050	360,724 / 1.875%	287 / 0.001%
Otti-000052	376,398 / 1.917%	236 / 0.001%
Otti-000051	430,366 / 2.104%	281 / 0.001%
Otti-000053	420,691 / 2.187%	222 / 0.001%
Otti-000054	350,916 / 2.057%	204 / 0.001%
Otti-000055	332,768 / 1.914%	226 / 0.001%
Otti-000056	347,766 / 1.786%	361 / 0.002%
Otti-000057	339,403 / 1.911%	249 / 0.001%
Otti-000059	302,443 / 1.74%	196 / 0.001%
Otti-000060	334,088 / 1.674%	220 / 0.001%
Otti-000058	261,569 / 1.413%	193 / 0.001%
Otti-000062	247,290 / 1.179%	143 / 0.001%
Otti-000042	87,801 / 0.421%	64 / 0%
Otti-000064	322,817 / 1.486%	277 / 0.001%
Otti-000061	197,159 / 0.904%	182 / 0.001%
Otti-000065	303,144 / 1.725%	261 / 0.001%
Otti-000066	292,780 / 1.605%	235 / 0.001%
Otti-000067	403,772 / 2.047%	252 / 0.001%
Otti-000069	284,505 / 1.848%	238 / 0.002%
Otti-000070	366,578 / 2.084%	193 / 0.001%
Otti-000068	246,956 / 1.416%	260 / 0.001%
Otti-000071	361,369 / 2.096%	230 / 0.001%
Otti-000043	81,809 / 0.336%	64 / 0%
Otti-000063	113,304 / 0.641%	100 / 0.001%
Otti-000073	375,226 / 1.917%	318 / 0.002%
Otti-000076	264,333 / 1.389%	259 / 0.001%
Otti-000077	320,440 / 1.882%	215 / 0.001%
Otti-000078	313,039 / 1.945%	213 / 0.001%
Otti-000079	250,946 / 1.421%	343 / 0.002%
Otti-000081	329,798 / 2.052%	155 / 0.001%
Otti-000082	330,527 / 1.795%	219 / 0.001%
Otti-000080	283,064 / 1.668%	190 / 0.001%
Otti-000074	181,294 / 1.077%	184 / 0.001%
Otti-000085	344,409 / 1.836%	236 / 0.001%
Otti-000084	279,285 / 1.495%	161 / 0.001%
Otti-000086	281,285 / 1.675%	215 / 0.001%
Otti-000087	245,875 / 1.416%	206 / 0.001%
Otti-000088	316,320 / 1.901%	191 / 0.001%
Otti-000089	360,040 / 1.654%	237 / 0.001%
Otti-000090	333,348 / 1.666%	216 / 0.001%
Otti-000075	116,099 / 0.609%	89 / 0%
Otti-000171	232,929 / 1.346%	353 / 0.002%
Otti-000072	76,561 / 0.355%	74 / 0%
Otti-000083	92,073 / 0.436%	74 / 0%

Chimeric Reads

Name	Chimeric reads
Otti-000019	0 / 0%

Otti-000021	0 / 0%
Otti-000022	0 / 0%
Otti-000020	0 / 0%
Otti-000025	0 / 0%
Otti-000023	0 / 0%
Otti-000024	0 / 0%
Otti-000026	0 / 0%
Otti-000027	0 / 0%
Otti-000028	0 / 0%
Otti-000029	0 / 0%
Otti-000030	0 / 0%
Otti-000031	0 / 0%
Otti-000032	0 / 0%
Otti-000033	0 / 0%
Otti-000034	0 / 0%
Otti-000035	0 / 0%
Otti-000036	0 / 0%
Otti-000037	0 / 0%
Otti-000038	0 / 0%
Otti-000039	0 / 0%
Otti-000040	0 / 0%
Otti-000041	0 / 0%
Otti-000044	0 / 0%
Otti-000045	0 / 0%
Otti-000046	0 / 0%
Otti-000047	0 / 0%
Otti-000049	0 / 0%
Otti-000050	0 / 0%
Otti-000052	0 / 0%
Otti-000051	0 / 0%
Otti-000053	0 / 0%
Otti-000054	0 / 0%
Otti-000055	0 / 0%
Otti-000056	0 / 0%
Otti-000057	0 / 0%
Otti-000059	0 / 0%
Otti-000060	0 / 0%
Otti-000058	0 / 0%
Otti-000062	0 / 0%
Otti-000042	0 / 0%
Otti-000064	0 / 0%
Otti-000061	0 / 0%
Otti-000065	0 / 0%
Otti-000066	0 / 0%
Otti-000067	0 / 0%
Otti-000069	0 / 0%
Otti-000070	0 / 0%
Otti-000068	0 / 0%
Otti-000071	0 / 0%
Otti-000043	0 / 0%
Otti-000063	0 / 0%
Otti-000073	0 / 0%

Otti-000076	0 / 0%
Otti-000077	0 / 0%
Otti-000078	0 / 0%
Otti-000079	0 / 0%
Otti-000081	0 / 0%
Otti-000082	0 / 0%
Otti-000080	0 / 0%
Otti-000074	0 / 0%
Otti-000085	0 / 0%
Otti-000084	0 / 0%
Otti-000086	0 / 0%
Otti-000087	0 / 0%
Otti-000088	0 / 0%
Otti-000089	0 / 0%
Otti-000090	0 / 0%
Otti-000075	0 / 0%
Otti-000171	0 / 0%
Otti-000072	0 / 0%
Otti-000083	0 / 0%

Unmapped Reads

Name	Reads Unmapped: Too many mismatches	Reads Unmapped: Too short	Reads Unmapped: Other
Otti-000019	0 / 0%	855,348 / 4.58%	0 / 0%
Otti-000021	0 / 0%	1,009,774 / 4.72%	0 / 0%
Otti-000022	0 / 0%	928,241 / 5.15%	0 / 0%
Otti-000020	0 / 0%	956,762 / 5.01%	0 / 0%
Otti-000025	0 / 0%	1,222,588 / 5.33%	0 / 0%
Otti-000023	0 / 0%	1,281,010 / 6.73%	0 / 0%
Otti-000024	0 / 0%	1,081,504 / 5.25%	0 / 0%
Otti-000026	0 / 0%	788,126 / 4.18%	0 / 0%
Otti-000027	0 / 0%	1,189,467 / 6.03%	0 / 0%
Otti-000028	0 / 0%	690,970 / 4.15%	0 / 0%
Otti-000029	0 / 0%	959,554 / 4.71%	0 / 0%
Otti-000030	0 / 0%	889,684 / 4.65%	0 / 0%
Otti-000031	0 / 0%	892,656 / 5.12%	0 / 0%
Otti-000032	0 / 0%	972,240 / 5%	0 / 0%
Otti-000033	0 / 0%	1,035,362 / 4.85%	0 / 0%
Otti-000034	0 / 0%	1,016,124 / 6.11%	0 / 0%
Otti-000035	0 / 0%	1,109,558 / 5.26%	0 / 0%

Otti-000036	0 / 0%	813,495 / 5.39%	0 / 0%
Otti-000037	0 / 0%	961,362 / 5.73%	0 / 0%
Otti-000038	0 / 0%	1,084,213 / 5.46%	0 / 0%
Otti-000039	0 / 0%	897,062 / 4.73%	0 / 0%
Otti-000040	0 / 0%	944,222 / 5.26%	0 / 0%
Otti-000041	0 / 0%	957,361 / 4.05%	0 / 0%
Otti-000044	0 / 0%	929,973 / 4.94%	0 / 0%
Otti-000045	0 / 0%	968,902 / 5.56%	0 / 0%
Otti-000046	0 / 0%	1,215,335 / 6.14%	0 / 0%
Otti-000047	0 / 0%	1,059,198 / 5.17%	0 / 0%
Otti-000049	0 / 0%	1,463,951 / 7.26%	0 / 0%
Otti-000050	0 / 0%	1,034,766 / 5.38%	0 / 0%
Otti-000052	0 / 0%	1,142,704 / 5.82%	0 / 0%
Otti-000051	0 / 0%	1,562,950 / 7.64%	0 / 0%
Otti-000053	0 / 0%	1,146,358 / 5.96%	0 / 0%
Otti-000054	0 / 0%	1,113,955 / 6.53%	0 / 0%
Otti-000055	0 / 0%	1,210,010 / 6.96%	0 / 0%
Otti-000056	0 / 0%	1,252,252 / 6.43%	0 / 0%
Otti-000057	0 / 0%	1,086,890 / 6.12%	0 / 0%
Otti-000059	0 / 0%	1,030,811 / 5.93%	0 / 0%
Otti-000060	0 / 0%	882,215 / 4.42%	0 / 0%
Otti-000058	0 / 0%	3,825,034 / 20.67%	0 / 0%
Otti-000062	0 / 0%	8,186,449 / 39.02%	0 / 0%
Otti-000042	0 / 0%	16,205,448 / 77.79%	0 / 0%
Otti-000064	0 / 0%	1,005,907 / 4.63%	0 / 0%
Otti-000061	0 / 0%	9,242,468 / 42.39%	0 / 0%
Otti-000065	0 / 0%	922,460 / 5.25%	0 / 0%
Otti-000066	0 / 0%	833,488 / 4.57%	0 / 0%
Otti-000067	0 / 0%	1,084,815 / 5.5%	0 / 0%
Otti-000069	0 / 0%	814,599 / 5.29%	0 / 0%
Otti-000070	0 / 0%	860,137 / 4.89%	0 / 0%

Otti-000068	0 / 0%	1,756,058 / 10.07%	0 / 0%
Otti-000071	0 / 0%	1,099,916 / 6.38%	0 / 0%
Otti-000043	0 / 0%	19,926,718 / 81.89%	0 / 0%
Otti-000063	0 / 0%	10,331,849 / 58.42%	0 / 0%
Otti-000073	0 / 0%	1,274,465 / 6.51%	0 / 0%
Otti-000076	0 / 0%	953,452 / 5.01%	0 / 0%
Otti-000077	0 / 0%	798,739 / 4.69%	0 / 0%
Otti-000078	0 / 0%	872,483 / 5.42%	0 / 0%
Otti-000079	0 / 0%	810,589 / 4.59%	0 / 0%
Otti-000081	0 / 0%	749,022 / 4.66%	0 / 0%
Otti-000082	0 / 0%	1,018,240 / 5.53%	0 / 0%
Otti-000080	0 / 0%	2,377,572 / 14.01%	0 / 0%
Otti-000074	0 / 0%	7,760,231 / 46.12%	0 / 0%
Otti-000085	0 / 0%	922,823 / 4.92%	0 / 0%
Otti-000084	0 / 0%	4,715,761 / 25.25%	0 / 0%
Otti-000086	0 / 0%	826,397 / 4.92%	0 / 0%
Otti-000087	0 / 0%	809,143 / 4.66%	0 / 0%
Otti-000088	0 / 0%	760,334 / 4.57%	0 / 0%
Otti-000089	0 / 0%	896,973 / 4.12%	0 / 0%
Otti-000090	0 / 0%	1,250,924 / 6.25%	0 / 0%
Otti-000075	0 / 0%	12,831,387 / 67.31%	0 / 0%
Otti-000171	0 / 0%	1,227,063 / 7.09%	0 / 0%
Otti-000072	0 / 0%	17,865,364 / 82.91%	0 / 0%
Otti-000083	0 / 0%	16,268,093 / 77%	0 / 0%

Analysis Parameters

Parameter	Value
Upstream Files Pattern	_R1_001
Downstream Files Pattern	_R2_001
Provide Annotations	false
2-pass Mapping	false
Sort by Coordinate	true
Min. Intron Length	20
Max. Intron Length	1000000
Max. Distance Between Mates	1000000
Max. # of Multiple Alignments	20
Max. # of Mismatches	999
Include Chimeric Alignments	false
Add Read Group Information	false
Save Splice Junctions	false
Save Unmapped Reads	false

References

- Dobin A., Davis CA., Schlesinger F., Drenkow J., Zaleski C., Jha S., Batut P., Chaisson M. and Gingeras TR. (2013). STAR: ultrafast universal RNA-seq aligner. *Bioinformatics (Oxford, England), 29*(1), 15-21.
- OmicsBox - Bioinformatics made easy. BioBam Bioinformatics (Version 3.0.25). March 3, 2019. www.biobam.com/omicsbox.
