# Supplementary material for: Diesel exhaust particles alter gut microbiome and gene expression in the bumblebee Bombus terrestris
Source: Ecol Evol. 2023 Jun 21;13(6):e10180. doi: 10.1002/ece3.10180 (PMC10283033; doi:10.1002/ece3.10180)
Supplement: Supplementary file 1 — Appendix S1. [file ECE3-13-e10180-s001.zip › RNAseq_3_count_table_report_gene_level.pdf]

Gene-level Quantification

Name: Counts

Input 1: Annotation File

10,587 genomic features of type 'gene' and grouped by the 'gene_id' attribute have been retrieved from 397 reference sequences present in the annotation file 'Bombus_terrestris.Bter_1.0.51.gff3'.

Reference sequence	Genomic features	Length (bp)
GL899452	2	7,397
GL900529	1	1,223
GL902949	1	995
GL903919	1	381
GL900525	1	567
GL900401	1	1,564
GL902950	1	1,208
GL899566	1	897
GL899322	2	6,408
GL899202	5	7,926
GL899565	1	2,464
GL899686	2	7,671
GL899447	1	1,549
GL899689	1	11,298
GL899580	1	9,825
GL899584	2	119,067
GL899101	1	8,668
GL899464	2	92,299
GL899340	1	1,355
GL899335	2	4,539
GL899578	76	1,299,707
GL899212	1	4,038
GL899575	2	8,477
GL899219	2	4,127
GL899337	1	1,611
GL899458	1	6,970
GL899579	1	1,884
GL899459	1	2,642
GL899232	1	1,805
GL902928	1	750
GL899230	1	2,547
GL899351	1	7,047
GL899110	1	4,964
GL899473	1	8,207
GL900627	1	1,964
GL902922	1	918
GL900623	1	585
GL902921	1	1,234
GL900750	1	1,819
GL899589	1	2,146
GL899465	1	26,835
GL899224	1	2,065
GL899587	104	2,237,278

GL899469	1	1,268
GL899107	1	5,336
GL899121	1	4,806
GL899363	3	4,688
GL902814	1	572
GL902815	1	942
GL900761	1	1,550
GL899358	1	3,221
GL899476	1	9,657
GL899597	1	3,019
GL899235	3	7,043
GL899477	1	4,994
GL899598	1	12,098
GL899239	2	9,200
GL899771	1	1,221
GL899892	2	3,931
GL899530	1	2,997
GL899893	1	6,337
GL900729	1	1,004
GL900609	1	1,569
GL900725	1	1,484
GL900963	1	752
GL899409	2	4,536
GL899649	1	3,112
GL899400	1	2,682
GL899763	1	2,412
GL899884	1	546
GL899526	1	1,824
GL899406	1	5,845
GL904094	1	1,863
GL899403	1	2,683
GL899404	1	8,054
GL899767	1	5,188
GL899540	1	4,152
GL899541	1	10,747
GL900734	1	1,112
GL901702	1	1,514
GL899533	1	20,020
GL899654	1	2,204
GL899534	2	29,984
GL899655	1	1,162
GL899774	1	2,134
GL899416	1	20,492
GL899414	1	565
GL899310	1	1,319
GL899431	2	97,145
GL899671	1	2,731
GL899429	1	6,432
GL899666	1	16,687
GL899300	1	4,672
GL899304	1	2,436
GL899789	1	4,610

GL899441	1	2,381
GL900837	1	1,062
GL899681	2	4,654
GL899561	6	178,392
GL899682	1	4,186
GL901923	1	891
GL899676	2	8,974
GL899314	2	3,486
GL899798	1	6,160
GL899795	2	4,581
GL899174	1	3,944
GL899178	2	8,545
GL899299	2	5,871
GL899297	1	13,372
GL900921	1	635
GL899290	1	5,646
GL903084	1	1,122
GL899289	1	4,748
GL899067	1	3,080
GL899065	1	2,188
GL899179	1	6,187
GL899074	1	3,929
GL899195	2	143,993
GL900903	1	1,872
GL899198	1	2,407
GL899191	1	3,458
GL899192	1	1,317
GL904158	1	1,648
GL904278	1	533
GL903189	1	1,251
GL904150	1	1,294
GL899084	1	1,602
GL899087	1	4,930
GL899088	1	4,993
GL900911	1	1,467
GL904043	1	529
GL899492	1	3,077
GL899130	1	3,129
GL899490	1	2,588
GL899370	1	2,914
GL899133	3	7,721
GL899496	1	170,677
GL899013	2	5,782
GL899255	1	2,260
GL899131	1	1,226
GL899373	1	3,049
GL899494	7	96,598
GL899011	1	20,456
GL899132	1	5,157
GL904258	1	754
GL899126	2	4,052
GL899127	1	7,492

GL899124	1	4,129
GL899245	1	2,115
GL903042	1	1,802
GL899488	82	1,539,683
GL899009	105	1,667,897
GL899261	1	5,862
GL899020	1	4,551
GL899380	1	4,992
GL899381	1	63,918
GL899265	1	3,485
GL899024	1	4,105
GL899145	2	5,891
GL899387	1	5,870
GL899263	1	3,278
GL899384	1	3,677
GL899264	1	1,336
GL899385	12	486,386
GL902089	1	954
GL904143	1	1,433
GL899016	1	3,555
GL899379	1	7,485
GL899259	1	5,285
GL899257	1	3,439
GL899030	1	23,910
GL899151	3	47,243
GL899270	1	2,562
B01	643	17,153,651
B02	593	13,603,873
GL899034	1	4,043
GL899397	1	21,990
B03	620	14,656,165
GL899156	1	3,558
GL899277	1	5,913
B04	549	14,241,696
B05	580	11,918,102
GL899154	1	2,399
B06	361	12,724,418
B07	740	18,145,390
B08	364	9,733,834
B09	654	15,655,298
GL899390	2	7,051
GL903145	1	666
GL899027	1	3,797
GL899268	1	3,041
B10	606	13,618,662
B11	804	17,228,712
GL899160	1	3,240
B12	555	12,868,931
GL899040	1	3,924
GL899282	1	3,465
B13	344	9,884,808
GL899166	1	2,187

B14	595	11,649,563
GL899288	1	2,078
B15	644	11,467,329
GL899285	1	3,278
B16	292	5,274,633
B17	166	3,558,169
B18	121	3,466,108
GL903034	1	918
GL899399	68	1,032,586
GL899279	1	4,332
GL902183	1	1,002
GL904215	1	1,096
GL903127	1	636
GL904213	1	936
GL901061	1	808
GL901182	1	1,490
GL898911	1	1,996
GL898910	1	6,388
GL898923	1	5,854
GL898925	1	2,917
GL903258	1	447
GL902042	1	1,109
GL902163	1	980
GL902162	1	1,083
GL904309	1	1,418
GL903459	1	514
GL903458	1	877
GL898934	2	5,337
GL902255	1	780
GL903344	1	775
GL898937	1	3,779
GL901168	1	1,399
GL901283	1	655
GL898938	1	1,999
GL899909	23	1,077,779
GL903460	1	1,012
GL899901	1	8,479
GL898932	1	8,073
GL904316	1	1,037
GL904204	1	929
GL898948	1	1,074
GL903116	1	1,654
GL902144	1	555
GL903112	1	652
GL903474	1	1,514
GL902380	1	936
GL901170	1	1,016
GL902140	1	961
GL898942	1	1,934
GL900049	1	1,339
GL899090	1	2,613
GL901139	1	1,477

GL899091	1	33,621
GL902349	1	1,702
GL903559	1	625
GL902596	1	1,313
GL900299	1	632
GL902477	1	1,022
GL902111	1	583
GL900054	1	2,947
GL902365	1	1,104
GL902362	1	466
GL903693	1	833
GL900026	1	2,260
GL903779	1	720
GL900275	1	587
GL902695	1	1,268
GL901003	1	696
GL902210	1	1,263
GL902452	1	1,259
GL902330	1	1,093
GL903782	1	1,496
GL900039	1	3,689
GL903427	1	842
GL901013	1	445
GL900047	1	1,754
GL902587	1	560
GL904401	1	501
GL898906	3	119,216
GL903671	1	734
GL898908	1	3,570
GL900042	1	2,076
GL900043	57	925,396
GL900008	1	2,632
GL900004	1	8,010
GL900007	1	2,585
GL899728	1	1,308
GL899608	1	2,457
GL899605	2	5,410
GL900255	1	1,005
GL900376	1	482
GL903523	1	661
GL899969	12	806,185
GL902791	1	543
GL899609	1	42,358
GL903761	1	926
GL901462	1	1,226
GL898872	1	32,746
GL898871	2	60,064
GL899842	5	108,741
GL898876	3	201,711
GL899845	1	3,502
GL898878	53	918,958
GL899601	1	2,645

GL899844	8	111,394
GL898890	15	361,134
GL899860	2	58,731
GL898892	1	2,880
GL902436	1	1,676
GL900022	2	6,654
GL901595	1	1,706
GL899738	1	2,763
GL902444	1	1,097
GL899731	1	4,680
GL899852	1	3,092
GL899973	1	1,901
GL899850	2	3,265
GL899730	1	3,187
GL898887	21	626,623
GL899977	1	6,436
GL899978	1	3,818
GL899854	4	42,440
GL899975	2	54,754
GL899613	1	13,702
GL899976	1	4,531
GL899991	1	2,613
GL899750	1	7,086
GL902407	1	1,447
GL902525	1	628
GL899629	1	6,510
GL902651	1	1,464
GL899748	1	2,258
GL903743	1	345
GL899628	5	421,143
GL902653	1	1,786
GL903863	1	730
GL900592	1	1,604
GL899500	1	4,084
GL899861	2	2,335
GL898895	19	933,213
GL899503	3	20,786
GL899987	18	510,902
GL903624	1	518
GL902537	1	1,144
GL900000	2	40,011
GL902663	1	1,568
GL902662	1	1,993
GL902302	1	819
GL899639	7	167,780
GL899632	1	3,250
GL899753	1	3,383
GL899995	1	1,330
GL899636	3	2,017
GL899757	1	2,491
GL899756	1	2,039
GL899805	1	4,663

GL899806	1	2,056
GL899924	1	2,213
GL900695	1	1,974
GL902991	1	1,736
GL899929	2	7,608
GL899802	1	7,104
GL903965	1	810
GL900578	1	1,111
GL903724	1	1,050
GL903845	1	840
GL899937	1	5,473
GL899817	1	6,274
GL903852	1	522
GL899939	1	68,141
GL903970	1	1,164
GL898963	1	7,015
GL898980	2	5,260
GL902848	1	462
GL898979	1	3,090
GL900671	1	597
GL899828	2	8,409
GL900673	1	1,205
GL898859	24	397,061
GL899826	1	2,112
GL900674	1	936
GL898856	18	344,124
GL899822	1	15,665
GL898991	1	4,006
GL900316	1	1,222
GL902616	1	1,218
GL901408	1	1,612
GL902617	1	1,609
GL902854	1	628
GL899959	1	4,260
GL899836	190	2,650,667
GL900443	1	559
GL899830	1	4,556
GL898863	2	80,028
GL898983	1	4,230
GL899835	2	3,392
GL899956	1	9,119
GL898867	5	130,679
**Total**	**10,587**	**238,654,322**

Input 2: Alignment Files

A total of 69 alignment files have been processed.

Sample	Filename	Program	Sequencing	Format
Otti-000049	Otti-000049.bam	STAR (2.7.8a), samtools (1.12)	Paired-End	bam
Otti-000088	Otti-000088.bam	STAR (2.7.8a), samtools (1.12)	Paired-End	bam
Otti-000082	Otti-000082.bam	STAR (2.7.8a), samtools (1.12)	Paired-End	bam
Otti-000081	Otti-000081.bam	STAR (2.7.8a), samtools (1.12)	Paired-End	bam

Otti-000084	Otti-000084.bam	STAR (2.7.8a), samtools (1.12)	Paired-End	bam
Otti-000040	Otti-000040.bam	STAR (2.7.8a), samtools (1.12)	Paired-End	bam
Otti-000056	Otti-000056.bam	STAR (2.7.8a), samtools (1.12)	Paired-End	bam
Otti-000080	Otti-000080.bam	STAR (2.7.8a), samtools (1.12)	Paired-End	bam
Otti-000058	Otti-000058.bam	STAR (2.7.8a), samtools (1.12)	Paired-End	bam
Otti-000057	Otti-000057.bam	STAR (2.7.8a), samtools (1.12)	Paired-End	bam
Otti-000034	Otti-000034.bam	STAR (2.7.8a), samtools (1.12)	Paired-End	bam
Otti-000078	Otti-000078.bam	STAR (2.7.8a), samtools (1.12)	Paired-End	bam
Otti-000035	Otti-000035.bam	STAR (2.7.8a), samtools (1.12)	Paired-End	bam
Otti-000079	Otti-000079.bam	STAR (2.7.8a), samtools (1.12)	Paired-End	bam
Otti-000036	Otti-000036.bam	STAR (2.7.8a), samtools (1.12)	Paired-End	bam
Otti-000037	Otti-000037.bam	STAR (2.7.8a), samtools (1.12)	Paired-End	bam
Otti-000030	Otti-000030.bam	STAR (2.7.8a), samtools (1.12)	Paired-End	bam
Otti-000074	Otti-000074.bam	STAR (2.7.8a), samtools (1.12)	Paired-End	bam
Otti-000031	Otti-000031.bam	STAR (2.7.8a), samtools (1.12)	Paired-End	bam
Otti-000059	Otti-000059.bam	STAR (2.7.8a), samtools (1.12)	Paired-End	bam
Otti-000053	Otti-000053.bam	STAR (2.7.8a), samtools (1.12)	Paired-End	bam
Otti-000052	Otti-000052.bam	STAR (2.7.8a), samtools (1.12)	Paired-End	bam
Otti-000055	Otti-000055.bam	STAR (2.7.8a), samtools (1.12)	Paired-End	bam
Otti-000054	Otti-000054.bam	STAR (2.7.8a), samtools (1.12)	Paired-End	bam
Otti-000050	Otti-000050.bam	STAR (2.7.8a), samtools (1.12)	Paired-End	bam
Otti-000019	Otti-000019.bam	STAR (2.7.8a), samtools (1.12)	Paired-End	bam
Otti-000051	Otti-000051.bam	STAR (2.7.8a), samtools (1.12)	Paired-End	bam
Otti-000171	Otti-000171.bam	STAR (2.7.8a), samtools (1.12)	Paired-End	bam
Otti-000090	Otti-000090.bam	STAR (2.7.8a), samtools (1.12)	Paired-End	bam
Otti-000067	Otti-000067.bam	STAR (2.7.8a), samtools (1.12)	Paired-End	bam
Otti-000023	Otti-000023.bam	STAR (2.7.8a), samtools (1.12)	Paired-End	bam
Otti-000068	Otti-000068.bam	STAR (2.7.8a), samtools (1.12)	Paired-End	bam
Otti-000024	Otti-000024.bam	STAR (2.7.8a), samtools (1.12)	Paired-End	bam
Otti-000076	Otti-000076.bam	STAR (2.7.8a), samtools (1.12)	Paired-End	bam
Otti-000069	Otti-000069.bam	STAR (2.7.8a), samtools (1.12)	Paired-End	bam
Otti-000032	Otti-000032.bam	STAR (2.7.8a), samtools (1.12)	Paired-End	bam
Otti-000025	Otti-000025.bam	STAR (2.7.8a), samtools (1.12)	Paired-End	bam
Otti-000077	Otti-000077.bam	STAR (2.7.8a), samtools (1.12)	Paired-End	bam
Otti-000063	Otti-000063.bam	STAR (2.7.8a), samtools (1.12)	Paired-End	bam
Otti-000033	Otti-000033.bam	STAR (2.7.8a), samtools (1.12)	Paired-End	bam
Otti-000026	Otti-000026.bam	STAR (2.7.8a), samtools (1.12)	Paired-End	bam
Otti-000039	Otti-000039.bam	STAR (2.7.8a), samtools (1.12)	Paired-End	bam
Otti-000064	Otti-000064.bam	STAR (2.7.8a), samtools (1.12)	Paired-End	bam
Otti-000038	Otti-000038.bam	STAR (2.7.8a), samtools (1.12)	Paired-End	bam
Otti-000020	Otti-000020.bam	STAR (2.7.8a), samtools (1.12)	Paired-End	bam
Otti-000071	Otti-000071.bam	STAR (2.7.8a), samtools (1.12)	Paired-End	bam
Otti-000070	Otti-000070.bam	STAR (2.7.8a), samtools (1.12)	Paired-End	bam
Otti-000045	Otti-000045.bam	STAR (2.7.8a), samtools (1.12)	Paired-End	bam
Otti-000073	Otti-000073.bam	STAR (2.7.8a), samtools (1.12)	Paired-End	bam
Otti-000021	Otti-000021.bam	STAR (2.7.8a), samtools (1.12)	Paired-End	bam
Otti-000022	Otti-000022.bam	STAR (2.7.8a), samtools (1.12)	Paired-End	bam
Otti-000065	Otti-000065.bam	STAR (2.7.8a), samtools (1.12)	Paired-End	bam
Otti-000089	Otti-000089.bam	STAR (2.7.8a), samtools (1.12)	Paired-End	bam
Otti-000027	Otti-000027.bam	STAR (2.7.8a), samtools (1.12)	Paired-End	bam
Otti-000066	Otti-000066.bam	STAR (2.7.8a), samtools (1.12)	Paired-End	bam
Otti-000047	Otti-000047.bam	STAR (2.7.8a), samtools (1.12)	Paired-End	bam

Otti-000029	Otti-000029.bam	STAR (2.7.8a), samtools (1.12)	Paired-End	bam
Otti-000046	Otti-000046.bam	STAR (2.7.8a), samtools (1.12)	Paired-End	bam
Otti-000028	Otti-000028.bam	STAR (2.7.8a), samtools (1.12)	Paired-End	bam
Otti-000085	Otti-000085.bam	STAR (2.7.8a), samtools (1.12)	Paired-End	bam
Otti-000061	Otti-000061.bam	STAR (2.7.8a), samtools (1.12)	Paired-End	bam
Otti-000041	Otti-000041.bam	STAR (2.7.8a), samtools (1.12)	Paired-End	bam
Otti-000060	Otti-000060.bam	STAR (2.7.8a), samtools (1.12)	Paired-End	bam
Otti-000086	Otti-000086.bam	STAR (2.7.8a), samtools (1.12)	Paired-End	bam
Otti-000042	Otti-000042.bam	STAR (2.7.8a), samtools (1.12)	Paired-End	bam
Otti-000062	Otti-000062.bam	STAR (2.7.8a), samtools (1.12)	Paired-End	bam
Otti-000087	Otti-000087.bam	STAR (2.7.8a), samtools (1.12)	Paired-End	bam
Otti-000043	Otti-000043.bam	STAR (2.7.8a), samtools (1.12)	Paired-End	bam
Otti-000044	Otti-000044.bam	STAR (2.7.8a), samtools (1.12)	Paired-End	bam

Results Overview

Input Reads		Aligned Reads					
**Sample**	**Total Records**	**Feature**	**No Feature**	**Ambiguous**	**Alignment not Unique**	**Low Alignment Quality**	**Not aligned**
Otti-000049	19,311,987	14,977,524 / 77.56%	2,090,845 / 10.83%	1,221,600 / 6.33%	1,021,250 / 5.29%	0	768 / 0.00%
Otti-000088	16,357,422	13,276,685 / 81.17%	1,277,906 / 7.81%	1,005,213 / 6.15%	797,079 / 4.87%	0	539 / 0.00%
Otti-000082	17,894,934	14,543,308 / 81.27%	1,408,506 / 7.87%	1,112,111 / 6.21%	830,393 / 4.64%	0	616 / 0.00%
Otti-000081	15,813,900	12,831,678 / 81.14%	1,203,287 / 7.61%	958,252 / 6.06%	820,022 / 5.19%	0	661 / 0.00%
Otti-000084	14,383,932	11,704,821 / 81.37%	1,111,212 / 7.73%	864,089 / 6.01%	703,323 / 4.89%	0	487 / 0.00%
Otti-000040	17,516,812	14,412,045 / 82.28%	1,316,984 / 7.52%	952,817 / 5.44%	834,038 / 4.76%	0	928 / 0.01%
Otti-000056	18,765,824	14,614,503 / 77.88%	1,992,487 / 10.62%	1,266,009 / 6.75%	891,471 / 4.75%	0	1,354 / 0.01%
Otti-000080	15,026,010	12,117,363 / 80.64%	1,305,576 / 8.69%	885,986 / 5.90%	716,173 / 4.77%	0	912 / 0.01%
Otti-000058	15,083,163	12,345,194 / 81.85%	1,211,084 / 8.03%	862,168 / 5.72%	664,339 / 4.40%	0	378 / 0.00%
Otti-000057	17,188,575	13,692,503 / 79.66%	1,563,816 / 9.10%	1,074,898 / 6.25%	856,590 / 4.98%	0	768 / 0.00%
Otti-000034	16,102,285	12,752,001 / 79.19%	1,447,989 / 8.99%	1,090,857 / 6.77%	810,137 / 5.03%	0	1,301 / 0.01%
Otti-000078	15,690,272	12,715,475 / 81.04%	1,234,300 / 7.87%	961,207 / 6.13%	778,661 / 4.96%	0	629 / 0.00%
Otti-000035	20,640,960	16,376,030 / 79.34%	1,901,684 / 9.21%	1,265,396 / 6.13%	1,096,861 / 5.31%	0	989 / 0.00%
Otti-000079	17,247,494	14,138,687 / 81.98%	1,412,990 / 8.19%	1,045,924 / 6.06%	649,284 / 3.76%	0	609 / 0.00%
Otti-000036	14,771,522	11,703,239 / 79.23%	1,321,866 / 8.95%	919,439 / 6.22%	826,135 / 5.59%	0	843 / 0.01%
Otti-000037	16,294,826	12,846,376 / 78.84%	1,516,472 / 9.31%	1,139,963 / 7.00%	790,890 / 4.85%	0	1,125 / 0.01%
Otti-000030	18,792,557	15,137,180 / 80.55%	1,556,237 / 8.28%	1,189,862 / 6.33%	908,565 / 4.83%	0	713 / 0.00%
Otti-000074	9,341,394	7,442,031 / 79.67%	884,127 / 9.46%	558,414 / 5.98%	456,278 / 4.88%	0	544 / 0.01%
Otti-000031	16,991,428	13,652,918 / 80.35%	1,574,464 / 9.27%	1,029,620 / 6.06%	733,659 / 4.32%	0	767 / 0.00%

Otti-000059	16,814,845	13,775,689 / 81.93%	1,295,574 / 7.70%	977,977 / 5.82%	765,001 / 4.55%	0	604 / 0.00%
Otti-000053	18,729,698	14,825,813 / 79.16%	1,653,512 / 8.83%	1,187,010 / 6.34%	1,062,422 / 5.67%	0	941 / 0.01%
Otti-000052	19,066,233	15,250,782 / 79.99%	1,706,390 / 8.95%	1,156,776 / 6.07%	951,714 / 4.99%	0	571 / 0.00%
Otti-000055	16,680,896	13,291,060 / 79.68%	1,496,157 / 8.97%	1,055,068 / 6.33%	838,030 / 5.02%	0	581 / 0.00%
Otti-000054	16,476,112	12,840,464 / 77.93%	1,700,043 / 10.32%	1,052,016 / 6.39%	882,690 / 5.36%	0	899 / 0.01%
Otti-000050	18,748,186	15,043,315 / 80.24%	1,582,682 / 8.44%	1,209,794 / 6.45%	911,372 / 4.86%	0	1,023 / 0.01%
Otti-000019	18,325,076	14,819,052 / 80.87%	1,520,550 / 8.30%	1,150,150 / 6.28%	834,415 / 4.55%	0	909 / 0.00%
Otti-000051	19,542,370	15,280,835 / 78.19%	2,008,017 / 10.28%	1,173,908 / 6.01%	1,078,631 / 5.52%	0	979 / 0.01%
Otti-000171	16,447,022	12,649,239 / 76.91%	2,233,814 / 13.58%	961,760 / 5.85%	601,133 / 3.65%	0	1,076 / 0.01%
Otti-000090	19,271,326	15,700,196 / 81.47%	1,521,766 / 7.90%	1,207,831 / 6.27%	840,728 / 4.36%	0	805 / 0.00%
Otti-000067	19,249,972	15,422,877 / 80.12%	1,636,189 / 8.50%	1,175,301 / 6.11%	1,015,018 / 5.27%	0	587 / 0.00%
Otti-000023	18,217,914	14,485,625 / 79.51%	1,779,144 / 9.77%	1,192,793 / 6.55%	759,481 / 4.17%	0	871 / 0.00%
Otti-000068	16,067,189	12,983,202 / 80.81%	1,378,598 / 8.58%	1,073,110 / 6.68%	631,378 / 3.93%	0	901 / 0.01%
Otti-000024	20,079,067	16,215,663 / 80.76%	1,728,674 / 8.61%	1,207,835 / 6.02%	926,130 / 4.61%	0	765 / 0.00%
Otti-000076	18,487,915	15,147,876 / 81.93%	1,663,631 / 9.00%	1,000,578 / 5.41%	675,304 / 3.65%	0	526 / 0.00%
Otti-000069	15,013,200	12,036,307 / 80.17%	1,381,061 / 9.20%	880,523 / 5.86%	714,573 / 4.76%	0	736 / 0.00%
Otti-000032	19,049,721	15,293,893 / 80.28%	1,634,206 / 8.58%	1,165,052 / 6.12%	955,661 / 5.02%	0	909 / 0.00%
Otti-000025	22,372,982	18,048,460 / 80.67%	1,884,704 / 8.42%	1,348,950 / 6.03%	1,089,580 / 4.87%	0	1,288 / 0.01%
Otti-000077	16,718,854	13,680,582 / 81.83%	1,222,822 / 7.31%	1,007,095 / 6.02%	807,338 / 4.83%	0	1,017 / 0.01%
Otti-000063	7,529,322	6,121,508 / 81.30%	699,468 / 9.29%	418,520 / 5.56%	289,616 / 3.85%	0	210 / 0.00%
Otti-000033	21,016,926	16,731,162 / 79.61%	1,799,050 / 8.56%	1,319,619 / 6.28%	1,166,420 / 5.55%	0	675 / 0.00%
Otti-000026	18,508,659	15,136,502 / 81.78%	1,543,204 / 8.34%	1,108,650 / 5.99%	719,894 / 3.89%	0	409 / 0.00%
Otti-000039	18,516,670	15,011,985 / 81.07%	1,570,023 / 8.48%	1,203,240 / 6.50%	730,701 / 3.95%	0	721 / 0.00%
Otti-000064	21,229,747	17,359,638 / 81.77%	1,685,344 / 7.94%	1,351,240 / 6.36%	832,975 / 3.92%	0	550 / 0.00%
Otti-000038	19,410,344	15,257,398 / 78.60%	1,823,269 / 9.39%	1,273,166 / 6.56%	1,055,677 / 5.44%	0	834 / 0.00%
Otti-000020	18,676,411	15,207,090 / 81.42%	1,483,820 / 7.94%	1,102,156 / 5.90%	882,534 / 4.73%	0	811 / 0.00%
Otti-000071	16,680,458	13,208,335 / 79.18%	1,491,420 / 8.94%	1,078,050 / 6.46%	901,362 / 5.40%	0	1,291 / 0.01%
Otti-000070	17,280,445	13,776,887 / 79.73%	1,466,626 / 8.49%	1,118,779 / 6.47%	917,391 / 5.31%	0	762 / 0.00%
Otti-000045	16,992,940	13,672,420 / 80.46%	1,389,633 / 8.18%	1,043,305 / 6.14%	886,990 / 5.22%	0	592 / 0.00%
Otti-000073	18,872,684	14,996,825 / 79.46%	1,794,455 / 9.51%	1,133,958 / 6.01%	946,636 / 5.02%	0	810 / 0.00%
Otti-000021	20,916,950	17,001,048 / 81.28%	1,870,022 / 8.94%	1,166,187 / 5.58%	879,099 / 4.20%	0	594 / 0.00%

Otti-000022	17,597,297	14,281,184 / 81.16%	1,453,642 / 8.26%	1,032,336 / 5.87%	829,197 / 4.71%	0	938 / 0.01%
Otti-000065	17,120,383	13,821,767 / 80.73%	1,468,684 / 8.58%	1,052,776 / 6.15%	776,272 / 4.53%	0	884 / 0.01%
Otti-000089	21,422,861	17,443,046 / 81.42%	1,835,607 / 8.57%	1,234,587 / 5.76%	908,819 / 4.24%	0	802 / 0.00%
Otti-000027	19,143,715	15,237,404 / 79.59%	1,689,852 / 8.83%	1,205,598 / 6.30%	1,010,119 / 5.28%	0	742 / 0.00%
Otti-000066	17,858,149	14,515,052 / 81.28%	1,416,382 / 7.93%	1,179,168 / 6.60%	746,857 / 4.18%	0	690 / 0.00%
Otti-000047	20,071,445	16,081,373 / 80.12%	1,735,814 / 8.65%	1,189,196 / 5.92%	1,064,412 / 5.30%	0	650 / 0.00%
Otti-000029	19,981,831	16,126,999 / 80.71%	1,657,783 / 8.30%	1,251,387 / 6.26%	944,838 / 4.73%	0	824 / 0.00%
Otti-000046	19,054,317	15,412,730 / 80.89%	1,647,668 / 8.65%	1,221,296 / 6.41%	771,667 / 4.05%	0	956 / 0.01%
Otti-000028	16,323,019	13,321,998 / 81.61%	1,328,186 / 8.14%	1,092,646 / 6.69%	579,439 / 3.55%	0	750 / 0.00%
Otti-000085	18,352,664	14,952,245 / 81.47%	1,445,410 / 7.88%	1,091,699 / 5.95%	862,755 / 4.70%	0	555 / 0.00%
Otti-000061	12,869,825	10,504,349 / 81.62%	1,080,319 / 8.39%	777,830 / 6.04%	506,960 / 3.94%	0	367 / 0.00%
Otti-000041	23,263,340	18,971,346 / 81.55%	1,928,972 / 8.29%	1,395,882 / 6.00%	966,299 / 4.15%	0	841 / 0.00%
Otti-000060	19,583,972	16,117,813 / 82.30%	1,548,540 / 7.91%	1,075,275 / 5.49%	841,763 / 4.30%	0	581 / 0.00%
Otti-000086	16,403,371	13,286,369 / 81.00%	1,385,053 / 8.44%	1,016,649 / 6.20%	714,341 / 4.35%	0	959 / 0.01%
Otti-000042	4,762,248	3,846,882 / 80.78%	408,773 / 8.58%	283,221 / 5.95%	223,209 / 4.69%	0	163 / 0.00%
Otti-000062	13,166,000	10,782,047 / 81.89%	1,015,361 / 7.71%	747,810 / 5.68%	620,391 / 4.71%	0	391 / 0.00%
Otti-000087	16,935,021	13,656,576 / 80.64%	1,660,770 / 9.81%	990,992 / 5.85%	626,095 / 3.70%	0	588 / 0.00%
Otti-000043	4,534,936	3,708,791 / 81.78%	367,694 / 8.11%	248,929 / 5.49%	209,345 / 4.62%	0	177 / 0.00%
Otti-000044	18,332,045	14,801,408 / 80.74%	1,636,749 / 8.93%	1,182,787 / 6.45%	710,471 / 3.88%	0	630 / 0.00%

Warnings:

- 673 features (6.36%) for which no aligned reads were detected in any of the samples. Features with low counts provide little evidence for differential expression so they should be filtered out to improve further analysis.

Analysis Parameters

Parameter	Value
Feature File	Bombus_terrestris.Bter_1.0.51.gff3
Quantification Level	gene
Group by	gene_id
Strand Specificity	Non Strand Specific
Overlap Mode	Union
Lowest Mapping Quality	10

References

- Anders S., Pyl PT. and Huber W. (2015). HTSeq--a Python framework to work with high-throughput sequencing data. *Bioinformatics (Oxford, England), 31*(2), 166-9.

- OmicsBox - Bioinformatics made easy. BioBam Bioinformatics (Version 2.0.36). March 3, 2019. www.biobam.com/omicsbox.
